# Podocyte RIPK3 Deletion Improves Diabetic Kidney Disease by Attenuating NF‐κB p65 Driven Inflammation

**DOI:** 10.1002/advs.202503325

**Published:** 2025-06-20

**Authors:** Lu'an Li, Jiaying Li, Ruizhao Li, Xingchen Zhao, Yuanhan Chen, Yating Cai, Yan Yang, Weiteng Wang, Siqi Zheng, Li Zhang, Xinling Liang

**Affiliations:** ^1^ Department of Nephrology Guangdong Provincial People's Hospital (Guangdong Academy of Medical Sciences) Southern Medical University Guangzhou 510080 China

**Keywords:** diabetic kidney disease, inflammation, NF‐κB p65, podocyte, RIPK3

## Abstract

Receptor‐interacting protein kinase 3 (RIPK3) is a key player in necroptosis and an emerging inflammation regulator, whose contribution to podocyte injury in diabetic kidney disease (DKD) remain unclear. Here, podocyte‐specific RIPK3‐knockout (KO) DKD mice and high glucose (HG) cultured mouse podocytes are used to elucidate the protective effects of podocyte RIPK3 deletion on DKD, explore the molecular pathogenic mechanisms of RIPK3 in podocyte injury, and assess pharmacological inhibition of RIPK3 signaling as a therapeutic strategy. The results demonstrated that podocyte‐specific RIPK3‐KO alleviated albuminuria, mesangial matrix proliferation, foot process fusion, and podocyte loss in DKD mice. Additionally, podocyte RIPK3 is upregulated in renal biopsies with DKD and expression is negatively correlated with albuminuria. In vitro, knockdown of RIPK3 using small interfering RNA (siRNA) or inhibition with GSK'872 prevented podocyte injury. RNA sequencing of mouse podocytes revealed that the knockdown of RIPK3 can alleviate HG‐induced activation of the NF‐κB‐related inflammatory pathways. Importantly, pharmacological inhibition of RIPK3 by GSK'872 alleviated podocyte damage, and reduced proteinuria in DKD mice. Overall, these results uncovered a novel role of podocyte RIPK3 in promoting podocyte injury and DKD progression by regulating NF‐κB‐mediated inflammatory signaling independent of necroptosis, offering novel insights and potential therapeutic strategies for DKD management.

## Graphical abstract

Prevention and treatment of diabetic kidney disease (DKD) have become a major global public health challenge. This study shed new light on the underlying pathogenic mechanisms of podocyte injury in DKD and highlights the therapeutic potential of mitigating podocyte injury through inhibition of the RIPK3/NF‐κB p65‐mediated inflammatory response as a viable treatment option for DKD.

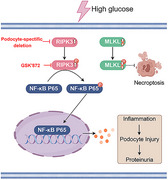

## 1 Introduction

Diabetic kidney disease (DKD) is the primary cause of end‐stage renal disease, accounting for ≈30%–40% of all cases.^[^ ^1^, ^2^ ^]^ Although various novel drugs have been developed for treatment of diabetes and DKD, the incidence and mortality rate of DKD remain high. Prevention and treatment of DKD have become a major global public health challenge. However, it is crucial to elucidate the key pathogenic mechanisms of DKD to identify new therapeutic targets.

Podocytes, as highly specialized terminally differentiated cells, constitute a critical component of the glomerular filtration barrier and play a pivotal role in the early disease progression of DKD.^[^ ^3^, ^4^ ^]^ Morphological changes, death, or loss of podocytes could lead to proteinuria and accelerate the decline of kidney function in DKD.^[^ ^5^, ^6^ ^]^ Recently identified mechanisms that contribute to podocyte injury in DKD include oxidative stress, inflammation, abnormal lipid metabolism, mitochondrial dysfunction, and autophagy dysfunction.^[^ ^7^, ^8^, ^9^, ^10^ ^]^ Despite advances in understanding the mechanisms of podocyte injury, further research is required to effectively ameliorate podocyte injury in DKD by specifically targeting one or more of these mechanisms.

Receptor interacting protein kinase‐3 (RIPK3), a serine/threonine kinase, is known to induce necroptosis, a form of programmed cell death that involves phosphorylation of mixed lineage kinase domain like ‌‌(MLKL).^[^ ^11^, ^12^ ^]^ Recent studies have shown that RIPK3 expression is upregulated in the podocytes of DKD patients and high glucose (HG) was shown to increase RIPK3 expression in podocytes in vitro.^[^ ^13^, ^14^ ^]^ However, these studies have not confirmed whether specific inhibition of podocyte RIPK3 can alleviate DKD in vivo. Although some studies have reported that HG induced necroptosis of podocytes in vitro,^[^ ^13^ ^]^ such pronounced morphological abnormalities are not characteristic pathological features of DKD. Previous studies have demonstrated that RIPK3 can regulate signaling pathways independent of necroptosis.^[^ ^15^, ^16^ ^]^ In this study, podocyte‐specific RIPK3‐knockout (KO) mice were employed to clarify the role of RIPK3 in DKD, combined with pharmacological targeting of RIPK3 to evaluate its therapeutic effects on DKD. Additionally, the molecular pathogenic mechanisms of RIPK3 in HG‐induced podocyte injury were investigated.

## 2 Results

### 2.1 Podocyte‐Specific KO of RIPK3 Mitigated Podocyte Injury in DKD Mice

**Figure 1:**
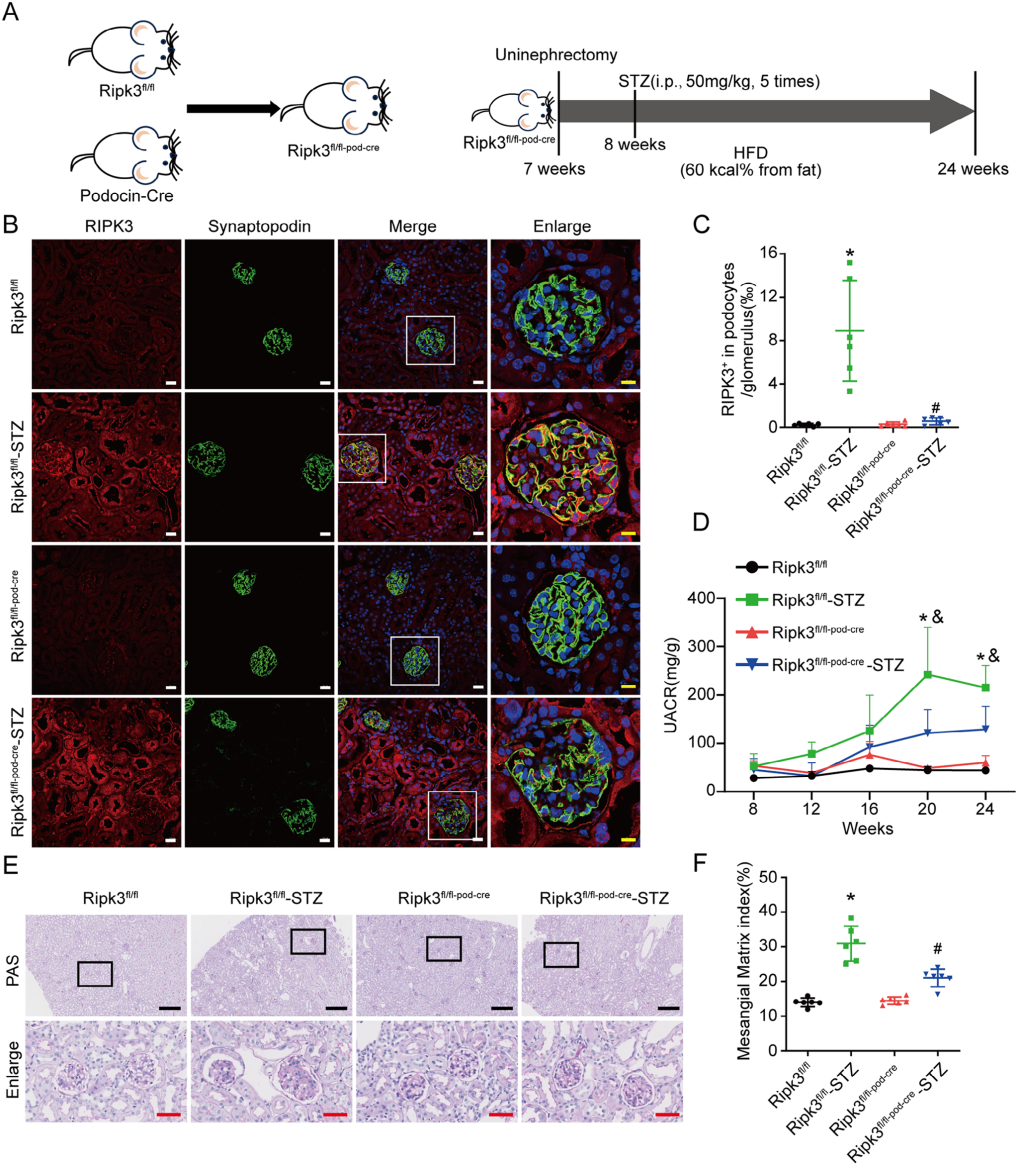
Podocyte‐specific KO of RIPK3 alleviated glomerulosclerosis and proteinuria in DKD mice. A) Experimental scheme for generation of Ripk3^fl/fl‐pod‐cre^ mice, and a schematic diagram showing the procedure of STZ‐induced diabetic mice. B,C) Representative immunofluorescence images and quantitative fluorescence analysis of RIPK3 (red) and synaptopodin (green) in kidney sections of mice (*n* = 6). Scale bars: white 20 µm, yellow 10 µm. D) Mouse UACR (*n* = 6) in mice. E) Morphological examination of glomerular changes in mice by PAS staining. Scale bars: black 250 µm, red 50 µm. F) Quantitative analysis of glomerular mesangial matrix expansion (*n* = 6) in mice. Data are shown as the mean ± SD. * versus Ripk3^fl/fl^, ^#^ versus Ripk3^fl/fl^‐STZ, ^&^ versus Ripk3^fl/fl‐pod‐cre^‐STZ, *p* < 0.05.

Podocyte‐specific RIPK3‐KO mice (Ripk3^fl/fl‐pod‐cre^) were generated using the Podocin‐Cre mediated Cre‐LoxP recombination system (**Figure** **1**A) and genotypes were confirmed by tail genotyping (Figure S1A, Supporting Information). Then, both Ripk3^fl/fl^ and Ripk3^fl/fl‐pod‐cre^ mice underwent uninephrectomy (UNI), followed by treatment with streptozotocin (STZ) and feeding a high‐fat diet (HFD) to induce DKD (referred to as Ripk3^fl/fl^‐STZ and Ripk3^fl/fl‐pod‐cre^‐STZ, respectively). As controls, sham surgery and a normal diet were administered to both mouse groups (referred to as Ripk3^fl/fl^ and Ripk3^fl/fl‐pod‐cre^, respectively). As compared to the Ripk3^fl/fl^ group, immunofluorescence and western blot analyses showed that RIPK3 expression was increased in both the glomerular and renal tubular cells of Ripk3^fl/fl^‐STZ mice, with notable co‐localization of RIPK3 and the podocyte marker protein synaptopodin (Figure 1B,C; Figure S1B‐D, Supporting Information). In contrast, as compared to the Ripk3^fl/fl^‐STZ mice, Ripk3^fl/fl‐pod‐cre^‐STZ mice exhibited reduced RIPK3 expression in podocytes, with no significant change to renal tubular RIPK3 expression (Figure 1B,C; Figure S1B–D, Supporting Information), indicating successful podocyte‐specific RIPK3 deletion.

**Figure 2:**
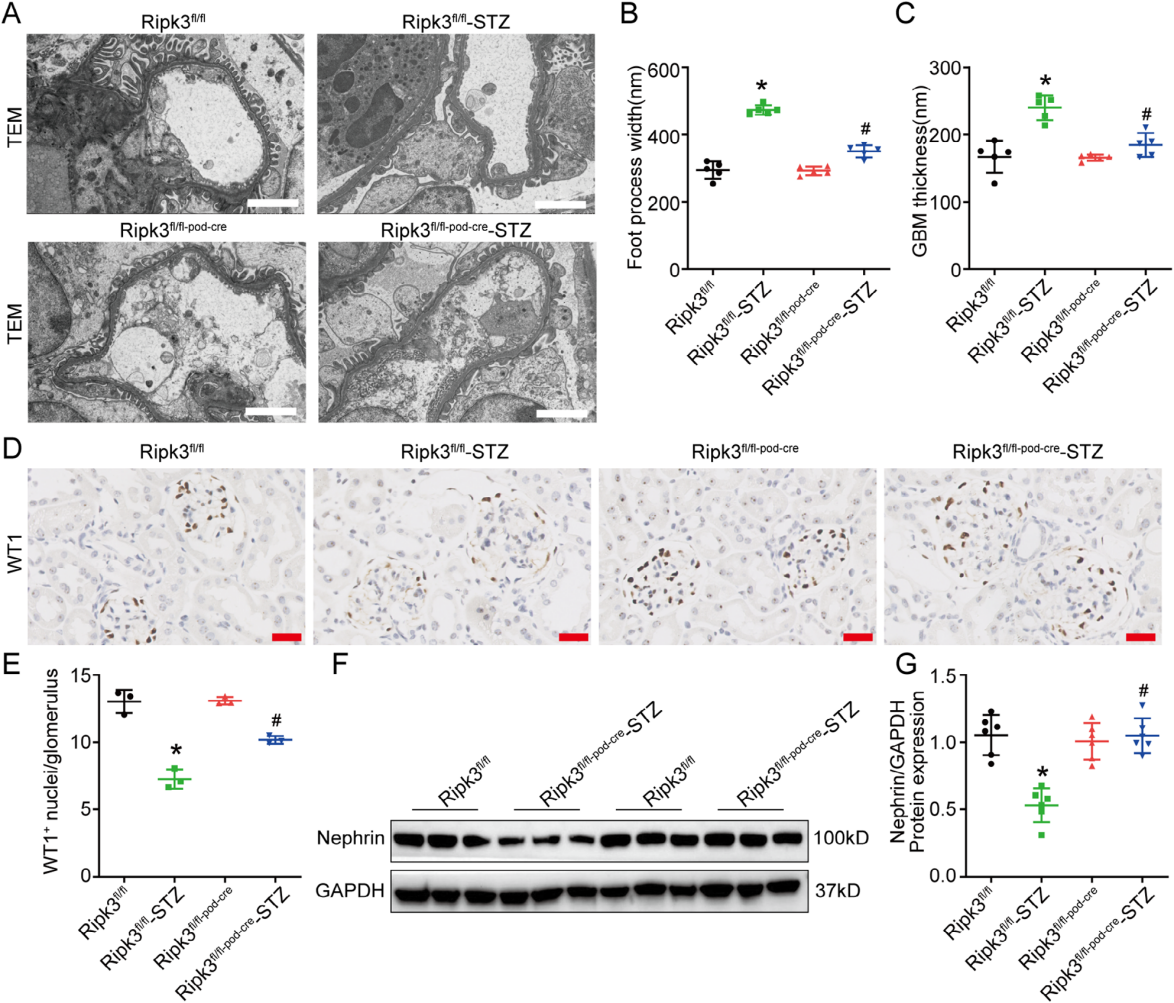
Podocyte‐specific KO of RIPK3 alleviated podocyte injury in DKD mice. A) Representative transmission electron micrographs (TEM) of the glomeruli in kidney sections of mice. Scale bars: white 2 µm. B,C) Quantification of foot process width (*n* = 3) and glomerular basement membrane (GBM) thickness (*n* = 3) in mice. D) Representative WT1–stained images of glomerulus in kidney sections of mice. Scale bars: red 25 µm. E) Quantification of WT1‐positive cells (*n* = 3) per glomerular section in mice. F,G) Representative western blot image and quantification of Nephrin (*n* = 6) in the renal cortex of mice. Data are shown as the mean ± SD. * versus Ripk3^fl/fl^, ^#^ versus Ripk3^fl/fl^‐STZ, *p* < 0.05.

At 16 weeks after induction of diabetes, the mice exhibited elevated blood glucose levels, increased kidney weight, and decreased body weight, while podocyte‐specific RIPK3 KO had no impact on these indicators (Figure S1E–I, Supporting Information). As compared to the Ripk3^fl/fl^‐STZ mice, the urine albumin‐to‐creatinine ratio (UACR) was lower in Ripk3^fl/fl‐pod‐cre^‐STZ mice (Figure 1D). Periodic acid‐Schiff (PAS) staining revealed that the Ripk3^fl/fl‐pod‐cre^‐STZ mice exhibited less glomerular mesangial matrix accumulation (Figure 1E–F). Electron microscopy showed decreased basement membrane thickening and podocyte foot process fusion in Ripk3^fl/fl‐pod‐cre^‐STZ mice (**Figure** **2**A–C). Furthermore, Wilms tumor protein‌‌ 1 (WT1) staining demonstrated that podocyte‐specific RIPK3 KO reduced podocyte loss caused by diabetes (Figure 2D,E). At the molecular level, western blot analysis showed that RIPK3 KO restored expression of the podocyte marker proteins nephrin and podocin (Figure 2F,G; Figure S1,K, Supporting Information). These results confirmed that RIPK3 was linked to podocyte injury in DKD mice.

### 2.2 RIPK3 was Significantly Elevated in the Podocytes of DKD Patients

**Figure 3:**
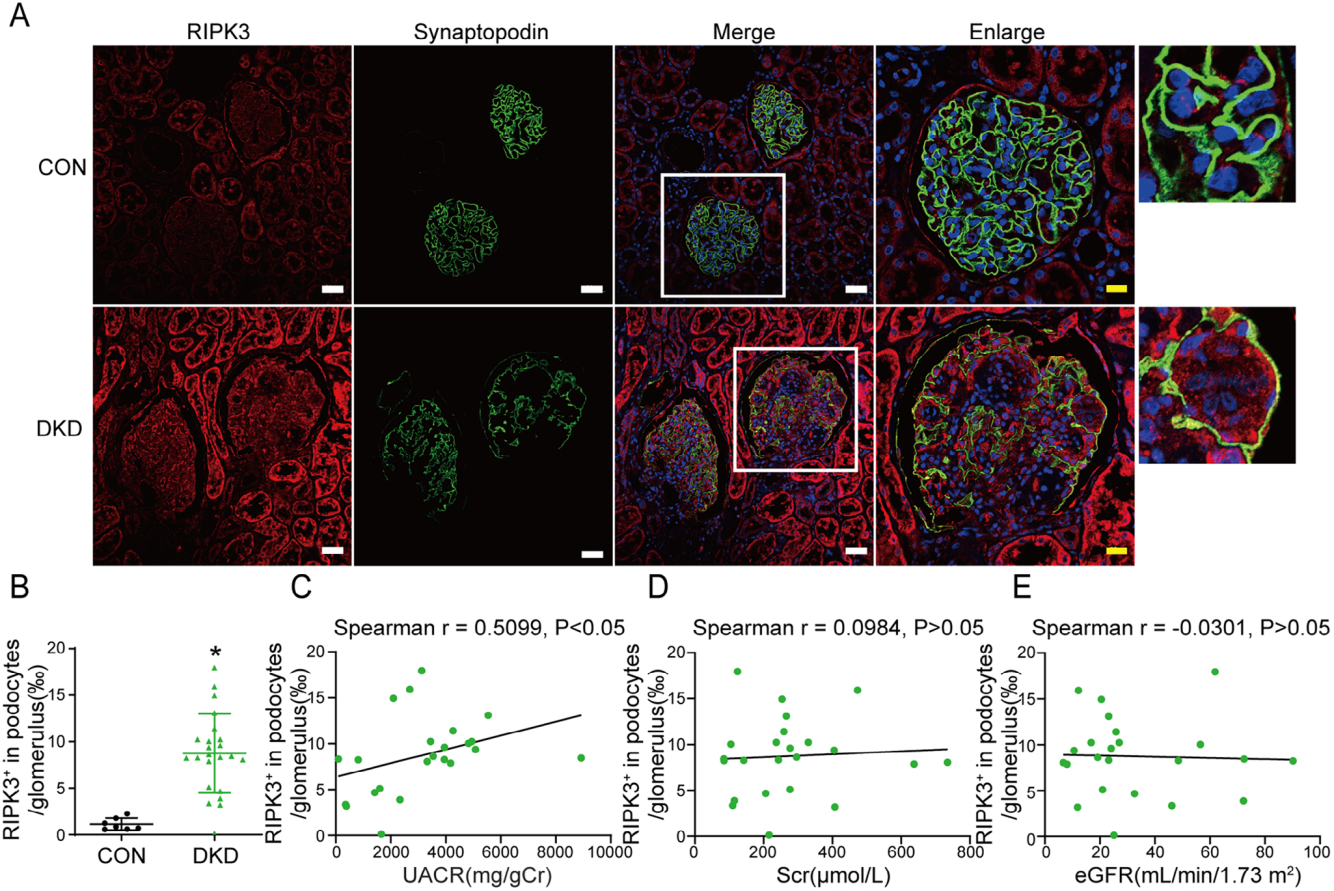
RIPK3 is significantly increased in podocytes of DKD patients. A) Representative immunofluorescence images of RIPK3 (red) and synaptopodin (green) in kidney sections of DKD patients and normal kidneys. Scale bars: white 50 µm, yellow 20 µm. B) Quantitative fluorescence analysis of RIPK3 in DKD (*n* = 23) and normal (*n* = 7) kidney sections. C,E) Correlation between RIPK3‐positive areas (RIPK3^+^) of podocytes/glomerulus and Scr, eGFR, and the UACR in all subjects (*n* = 23). Data are shown as the mean ± SD. * versus CON, *p* < 0.05.

Kidney specimens from 23 DKD patients were pathologically compared to adjacent normal kidney tissue specimens from seven patients with renal cell carcinoma. As compared to the control group, RIPK3 expression in podocytes, co‐localized with synaptopodin, was significantly increased in the DKD specimens (**Figure** **3**A,B). In addition, RIPK3 expression was also increased in the glomerular and renal tubular cells of DKD patients (Figure 3A). Furthermore, RIPK3 levels in podocytes were positively correlated with the UACR (Figure 3C), but not serum creatinine (Scr) levels (Figure 3D) or the estimated glomerular filtration rate (eGFR) (Figure 3E). These results suggest that RIPK3 may be involved in the process of podocyte injury in DKD.

### 2.3 The Role of RIPK3 in HG‐Induced Podocyte Injury In Vitro

**Figure 4:**
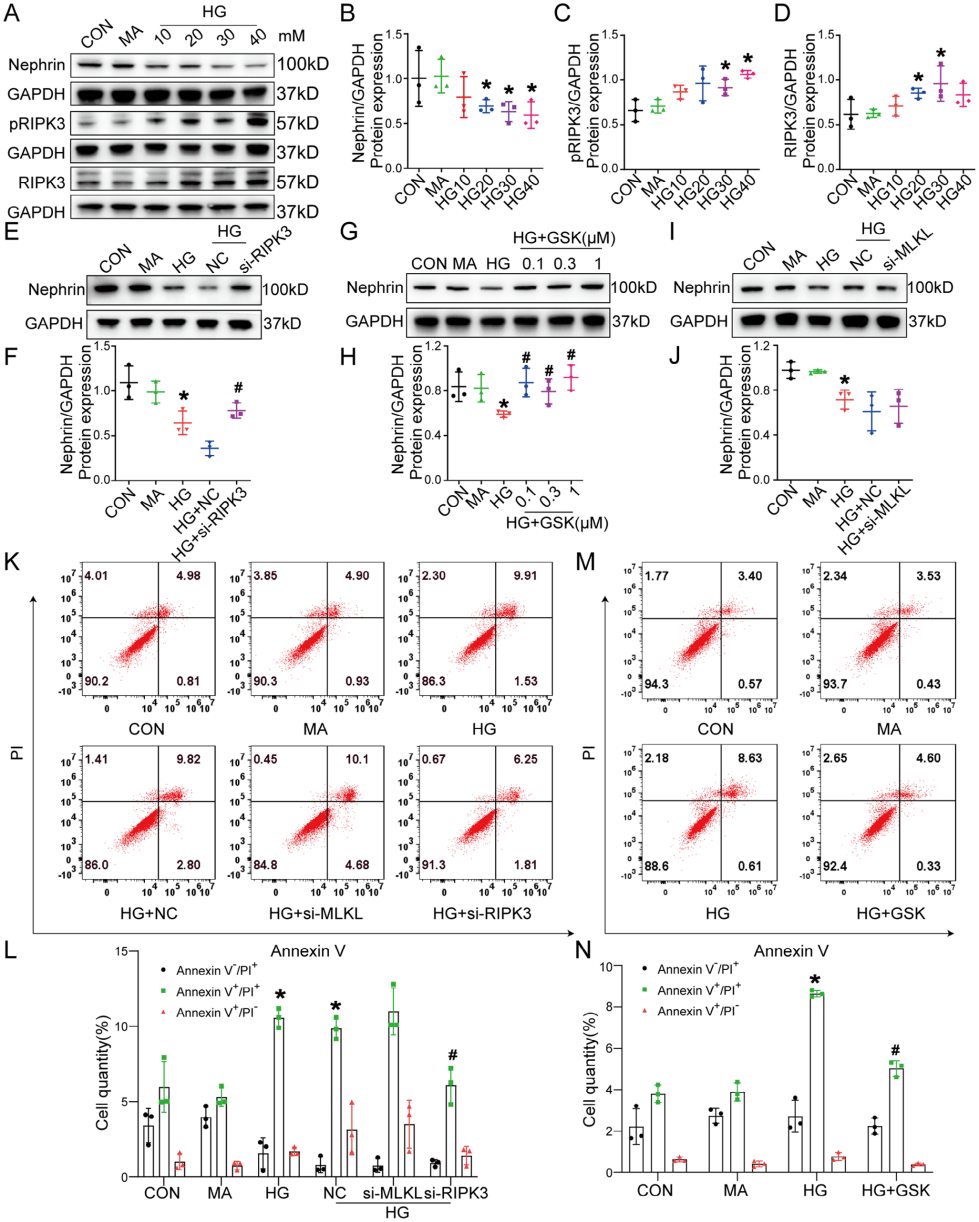
RIPK3, but not MLKL, was involved in HG‐induced podocyte injury. A,D) Representative western blot images and quantification of nephrin, pRIPK3 and RIPK3 in podocytes (*n* = 3) treated with varying concentrations of HG (10, 20, 30, and 40 mM) for 72 h. E,F) Representative western blot image and quantification of nephrin in podocytes (*n* = 3) treated with RIPK3‐targeting siRNA. G,H) Representative western blot image and quantification of nephrin in podocytes (*n* = 3) treated with GSK’872 (GSK). I,J) Representative western blot image and quantification of nephrin in podocytes (*n* = 3) treated with MLKL‐targeting siRNA. K,L) Flow cytometry analysis to assess the regulation of podocyte death by knockdown of RIPK3 and MLKL under HG treatment (*n* = 3). M,N) Flow cytometry analysis to assess the regulation of podocyte death by inhibiting RIPK3 (*n* = 3). Data are shown as the mean ± SD.* versus MA, ^#^ versus HG and HG+NC, *p* < 0.05.

To further investigate the role of RIPK3 in podocyte injury in DKD, podocytes were exposed to HG at concentrations of 10, 20, 30, and 40 mmol for 72 h to establish an in vitro model. Western blot analysis showed that as HG concentrations were increased, the expression of the podocyte marker proteins had progressively decreased (**Figure** **4**A,B; Figure S2A,B, Supporting Information), while expression of RIPK3 and phospho‐RIPK3 (pRIPK3) had increased, peaking at 30 mmol (Figure 4A,C,D). To further assess the role of RIPK3, small interfering RNA (siRNA) was employed for knockdown of RIPK3 mRNA expression (Figure S2C, Supporting Information) and GSK'872 was used to inhibit RIPK3 kinase activity. The immunoblotting results indicated that both knockdown and inhibition of RIPK3 significantly reversed the decreased expression of the podocyte marker proteins induced by HG (Figure 4E–H; Figure S2D–I, Supporting Information). Flow cytometry further demonstrated that both strategies reduced HG‐induced podocyte death (Figure 4K–N). These findings further confirmed that RIPK3 promoted HG‐induced podocyte injury.

### 2.4 RIPK3 Activated the NF‐κB p65 Pathway of Podocytes in DKD

RIPK3 is known to phosphorylate MLKL to induce necroptosis. However, no histologic evidence of necroptosis in DKD was shown by PAS staining and electron microscopy in this study. The protein expression levels of MLKL and pMLKL were not increased in the HG‐induced podocyte (Figure S2K–N, Supporting Information). MLKL knockdown (Figure S2J, Supporting Information) did not reverse the decrease in podocyte marker protein nephrin and synaptopodin expression (Figure 4I,J; Figure S2K, N, Supporting Information) or prevent HG‐induced podocyte death (Figure 4K,L), indicating that necroptosis pathway was not involved in podocyte injury of DKD.

**Figure 5:**
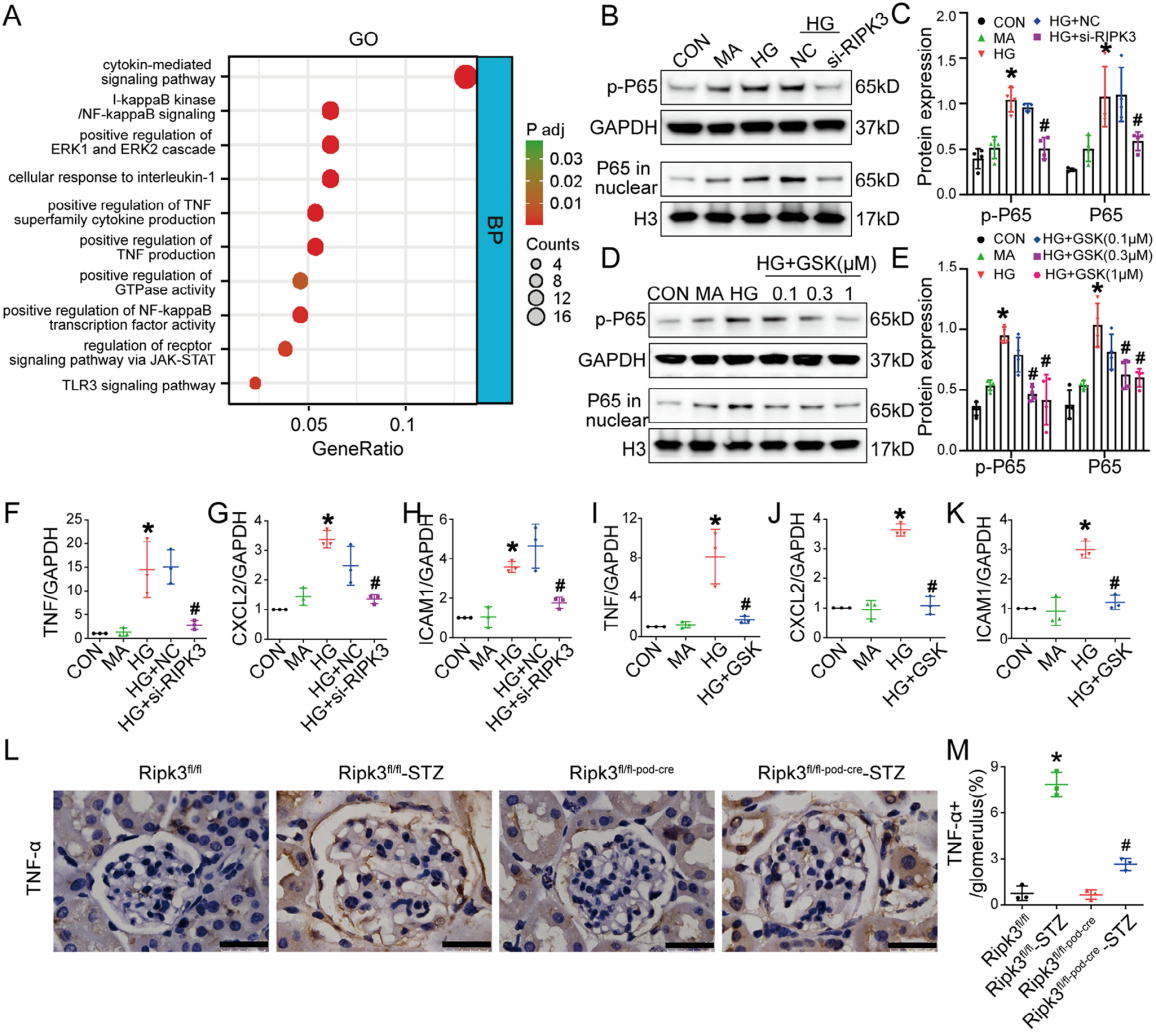
RIPK3 promoted the activation of the NF‐κB p65 inflammatory pathway in podocytes of DKD. A) GO pathway analysis. B,C) Representative western blot image and quantification of p‐p65 (*n* = 3) and p65 in nuclei (*n* = 3) of podocytes treated with RIPK3‐targeting siRNA. D,E) Representative western blot image and quantification of p‐p65 (*n* = 3) and p65 in the nuclei (*n* = 3) in podocytes treated with GSK’872(GSK). F–H) Relative mRNA levels of TNF, ICAM1, and CXCL2 in podocytes (*n* = 3) treated with RIPK3‐targeting siRNA. I–K) Relative mRNA levels of TNF, ICAM1, and CXCL2 in podocytes (*n* = 3) treated with GSK’872. L,M) Representative immunohistochemistry image and quantification of TNF‐α (*n* = 3) in kidney sections of mice. Scale bars: black 25 µm. Data are shown as the mean ± SD. * versus MA and Ripk3^fl/fl^, ^#^ versus HG, HG+NC and Ripk3^fl/fl^‐STZ, *p* < 0.05.

To further clarify the mechanism of RIPK3 to accelerate podocyte injury in the progression of DKD, whole transcriptome sequencing of podocyte samples from the CON, MA, HG, HG+NC, and HG+siRIPK3 groups was performed (Figure S3A, Supporting Information). As compared to the MA group, 1039 and 281 genes were upregulated and downregulated, respectively, in the HG group, while 69 and 240 genes were upregulated and downregulated, respectively, in the HG+siRIPK3 as compared to the HG+NC group. Intersection analysis of the upregulated genes between the HG and MA groups, in addition to the downregulated genes between the HG+siRIPK3 and HG+NC groups, revealed 169 overlapping genes (Figure S3B, Supporting Information). In contrast, intersection analysis of downregulated genes between the HG and MA groups, in addition to the upregulated genes between the HG+siRIPK3 and HG+NC groups, revealed only four overlapping genes (Figure S3B, Supporting Information). Considering the requirements for the number of genes in enrichment analysis, we subsequently proceeded to analyze only those 169 genes. There was no significant enrichment of the necroptosis pathway, as determined by Gene Ontology (GO) and Kyoto Encyclopedia of Genes and Genomes (KEGG) enrichment analyses, but there was significant enrichment in inflammatory pathways, with the nuclear factor kappa‐B (NF‐κB) signaling pathway as the most prominent (**Figure** **5**A; Figures S3C and S4A, Supporting Information). Both inhibition and knockdown of RIPK3 reduced protein expression of p‐NF‐κB p65 (p‐p65, Ser 536) and nuclear translocation of NF‐κB p65 (p65) with the in vitro HG model (Figure 5B–E). Real‐time quantitative polymerase chain reaction (RT‐qPCR) revealed that inhibition and knockdown of RIPK3 in vitro decreased expression of the inflammatory factors tumor necrosis factor (TNF), intercellular cell adhesion molecule 1 (ICAM1), and chemokine (C‐X‐C Motif) ligand 2 (CXCL2) (Figure 5F–K). Similarly, in vivo, RIPK3 KO also reduced TNF‐α protein levels in glomeruli (Figure 5L,M). Furthermore, luciferase reporter assays confirmed that RIPK3 directly enhances the transcriptional activity of NF‐κB (Figure S4B, Supporting Information), and co‐immunoprecipitation demonstrated an interaction between RIPK3 and NF‐κB p65 (Figure S4C,D). These findings suggest that RIPK3 may induce downstream inflammatory effects by promoting NF‐κB p65 phosphorylation in podocytes of DKD.

### 2.5 RIPK3 Promotes Podocyte Injury Through Activation of the NF‐κB p65 Pathway

To confirm the role of the NF‐κB p65 inflammatory signaling pathway in HG‐induced podocyte damage in vitro, the p65 inhibitor JSH‐23 and siRNA were utilized to suppress p65 activity (Figure S5A,B, Supporting Information). RT‐qPCR analysis revealed that both inhibition and knockdown of p65 significantly reduced expression of the inflammatory factors TNF, ICAM1, and CXCL2 in HG‐induced podocyte injury (Figure S5C–E, Supporting Information). Western blot analysis showed that both p65 inhibition and knockdown markedly restored the HG‐induced downregulation of the podocyte marker protein nephrin (Figure S5F,G, Supporting Information). Furthermore, flow cytometry indicated that inhibition and knockdown of p65 decreased HG‐induced podocyte death (Figure S5H,I, Supporting Information). These results confirmed that activation of the NF‐κB p65 pathway exacerbated HG‐induced podocyte injury.

**Figure 6:**
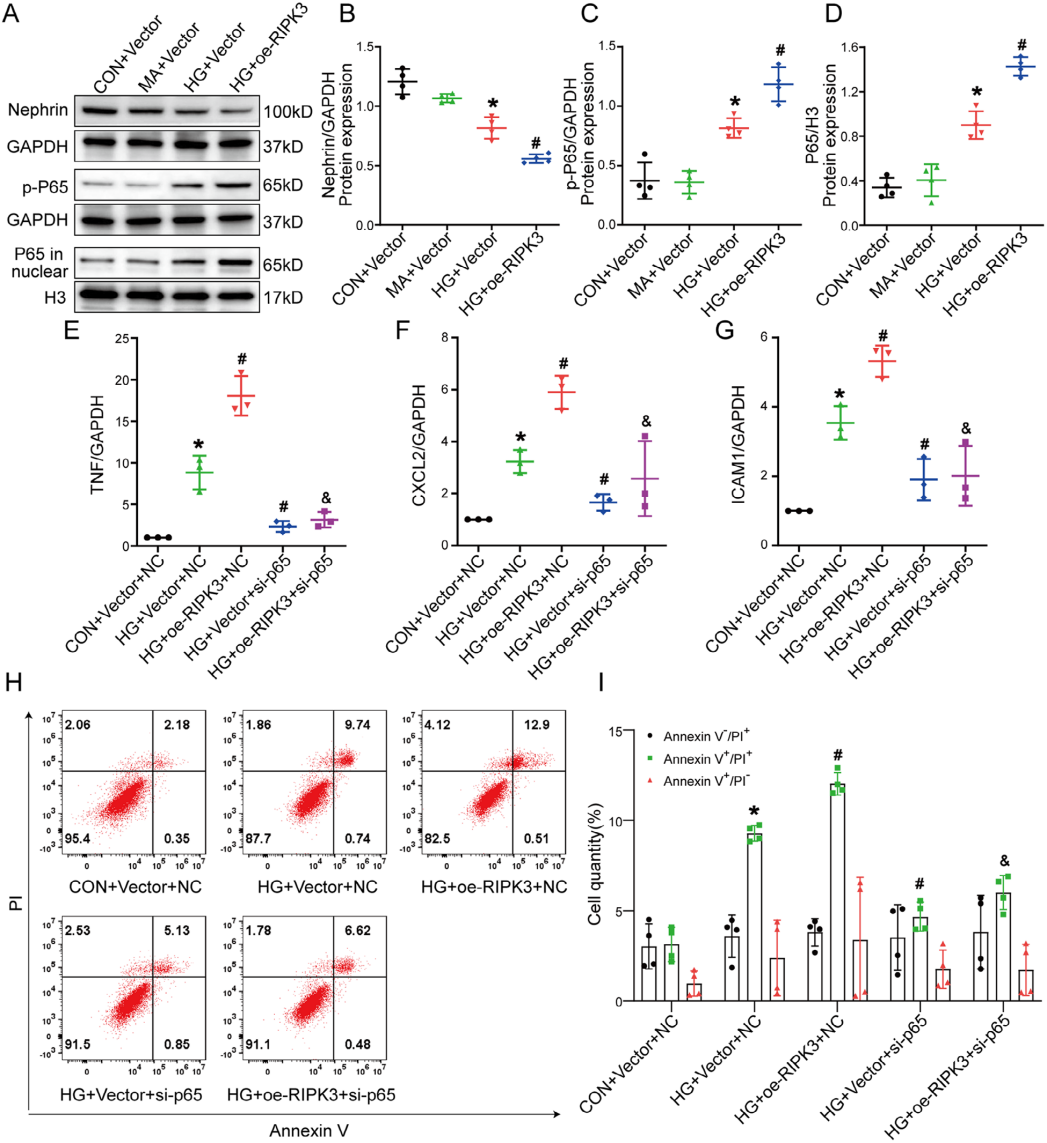
RIPK3 promoted podocyte injury through activation of the NF‐κB P65 pathway. Overexpression of RIPK3 in podocytes under HG conditions, followed by the addition of NF‐κB p65‐targeting siRNA. A‐D) Representative western blot images and quantification of nephrin, pRIPK3, and RIPK3 in podocytes (*n* = 4). E–G) Relative mRNA levels of TNF, ICAM1, and CXCL2 in podocytes (*n* = 3). H,I) Flow cytometry analysis to assess the regulation of podocyte death (*n* = 4). Data are shown as the mean ± SD. * versus MA+Vector and CON+Vector+NC, ^#^ versus HG+Vector and HG+Vector+NC, ^&^ versus HG+oe‐RIPK3+NC, *p* < 0.05.

To further determine whether RIPK3 promotes podocyte injury through activation of the NF‐κB p65 pathway, lentiviral vectors overexpressing RIPK3 were employed to establish podocyte injury model with RIPK3 overexpression under HG stimulation (Figure S6A–C, Supporting Information). The results revealed that, as compared to the HG group, overexpression of RIPK3 led to significant upregulation of p‐p65 and nuclear p65 in podocytes (**Figure** **6**A–D). Similarly, expression of inflammatory factors and cell death were significantly increased in HG‐treated podocytes overexpressing RIPK3 as compared to the HG group (Figure 6E–I). Conversely, knockdown of p65 by siRNA prevented the increased expression of inflammatory factors and cell death in the HG+oe‐RIPK3 group (Figure 6E–I). Further results were obtained with HG‐treated RIPK3‐knockdown podocytes after stimulation with NF‐κB activator 1 (Figure S6D–J, Supporting Information). These findings suggest that RIPK3 promotes podocyte injury through the activation of the p65 pathway.

### 2.6 RIPK3 Inhibition Alleviated Podocyte Injury in DKD Mice

**Figure 7:**
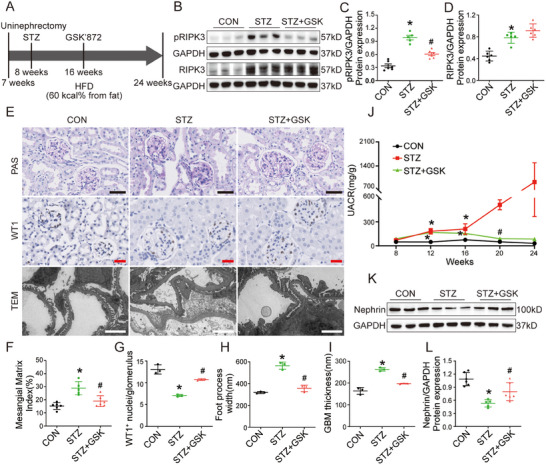
GSK’872(GSK) protected against DKD in vivo. A) A schematic diagram showing the procedure to generate STZ‐induced diabetic mice and intraperitoneal injection of GSK’872. B–D) Representative western blot images and quantification of pRIPK3 and RIPK3 in the renal cortex of mice (*n* = 5–6). E) Representative PAS staining, TEM, and WT1–stained images of glomeruli in kidney sections of mice. Scale bars: black 50 µm, red 25 µm, white 2 µm. F–I) Quantitative analysis of glomerular mesangial matrix expansion (*n* = 5–6), foot process width (*n* = 3), GBM thickness (*n* = 3), and WT1‐positive cells (*n* = 3) in mice. (J) UACR of mice (*n* = 5–6). (K‐L) Representative western blot image and quantification of nephrin (*n* = 5–6) in the renal cortex of mice. Data are shown as the mean ± SD. * versus CON, ^#^ versus STZ, *p* < 0.05.

To further investigate the role of RIPK3 as a molecular target for podocyte injury in DKD treatment, the STZ/HFD/UNI and db/db mice were administered the RIPK3 kinase inhibitor GSK'872 (**Figure** **7**A). The results showed that GSK'872 had no effect on blood glucose levels, kidney weight, or body weight (Figures S7A–D and S8A–D, Supporting Information). Western blot analysis showed that GSK'872 did not alter protein expression of RIPK3 in the kidney, but did reduce pRIPK3 levels (Figure 7B–D; Figure S8G–I, Supporting Information). Treatment with GSK'872 significantly lowered the UACR (Figures 7J and S8E, Supporting Information) and improved glomerular mesangial matrix accumulation (Figure 7E,F; Figure S8J,K, Supporting Information), basement membrane thickening (Figure 7E,I; Figure S8J, N, Supporting Information), and podocyte foot process fusion (Figure 7E,H; Figure S8J, M, Supporting Information). GSK'872 also restored the reduced podocyte count in the DKD mouse models (Figure 7E,G; Figure S8J, L, Supporting Information). Immunoblotting analysis revealed that the expression of the glomerular podocyte marker proteins nephrin and podocin was restored (Figure 7K,L, Figures S7F,G and S8O–Q, Supporting Information). Additionally, GSK'872 significantly suppressed both pNF‐κB p65 protein levels and transcriptional activation of inflammatory cytokines (Figure S9, Supporting Information). These findings indicate that the RIPK3 kinase inhibitor GSK'872 could attenuate podocyte injury of DKD mice by suppressing the activation of the NF‐κB p65 inflammatory pathway.

**Figure 8:**
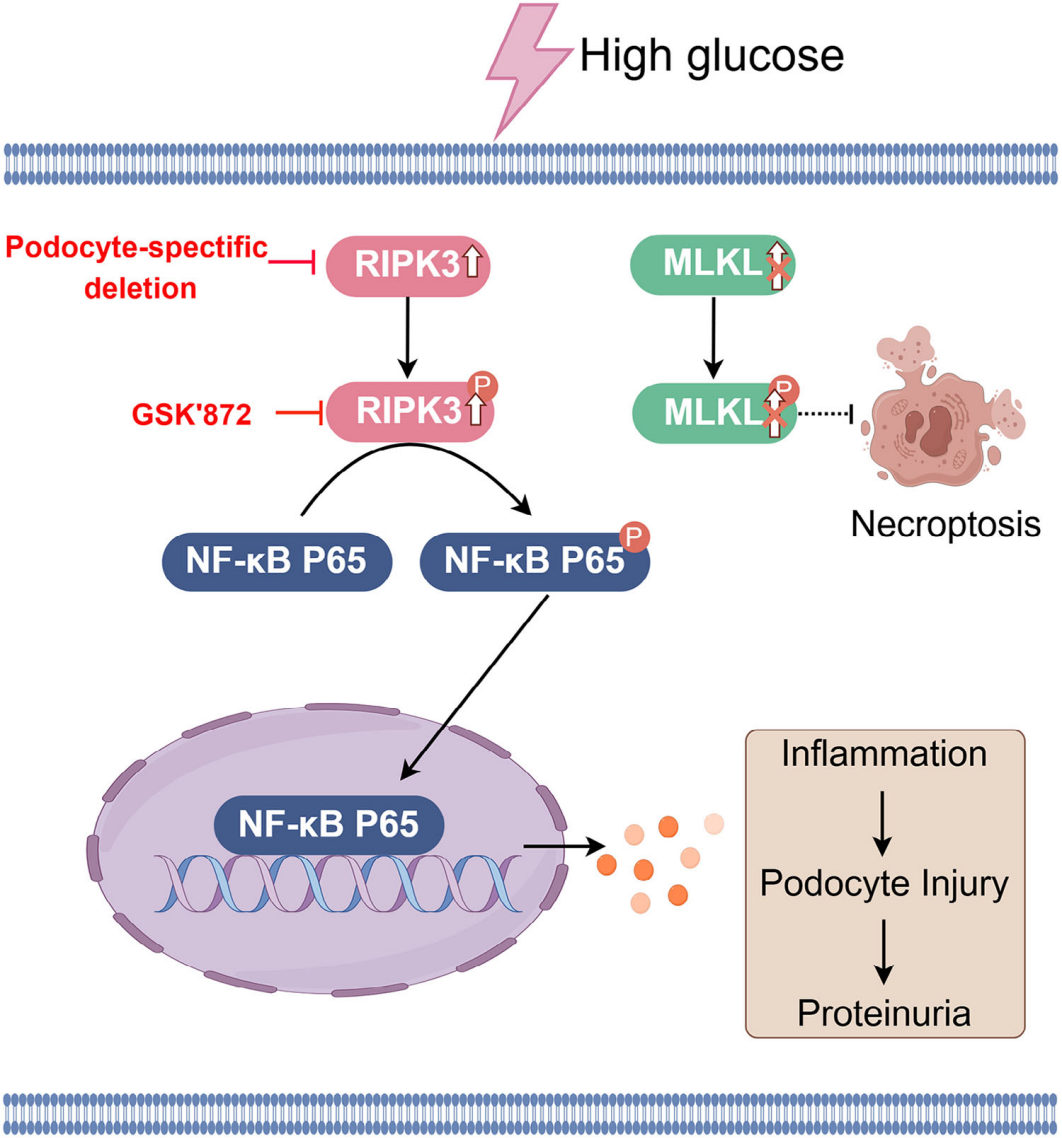
Schematic illustration of podocyte RIPK3 in the development of DKD.

## 3 Discussion

Podocyte injury plays a critical role in the development of proteinuria in DKD. Expanding current knowledge of the molecular mechanisms involved in podocyte injury may offer new opportunities to identify novel therapeutic targets for DKD. In this study, elevated RIPK3 levels promoted podocyte injury and albuminuria in DKD mice, which involved activation of the NF‐κB p65‐mediated inflammatory response, but not necroptosis. Importantly, pharmacological inhibition of RIPK3 has a renal protective effect, providing new insights into potential therapeutic strategies.

Previous studies have shown that RIPK3 is involved in HG‐induced podocyte injury in vitro.^[^ ^13^, ^14^ ^]^ Besides, a recent study using RIPK3‐KO mice with HFD‐induced diabetes demonstrated that RIPK3 KO alleviated podocyte injury and proteinuria.^[^ ^17^ ^]^ However, since RIPK3 is widely expressed and elevated in the glomeruli and renal tubules of DKD patients,^[^ ^17^, ^18^ ^]^ the renoprotective effects did not exclude the influence of other cell types. Additionally, a HFD induced only mild renal pathological changes,^[^ ^17^ ^]^ limiting observations of the actual impact of RIPK3 during DKD progression. In the present study, podocyte‐specific RIPK3‐KO mice and STZ‐induced DKD mice, combined with HFD feeding and UNI, were used to exacerbate glomerular sclerosis and podocyte injury.^[^ ^8^ ^]^ The results showed that podocyte‐specific RIPK3 KO significantly reduced glomerular mesangial matrix proliferation, foot process fusion, and podocyte loss, ultimately leading to reduced proteinuria, confirming the role of RIPK3 in podocyte injury of DKD. Previous studies have shown that RIPK3 expression is elevated in the glomeruli and renal tubules of patients with DKD,^[^ ^17^, ^18^ ^]^ and a portion of the elevated RIPK3 in the glomeruli can co‐localize with the podocyte marker protein synaptopodin.^[^ ^13^ ^]^ Meanwhile, the results of the present study found that podocyte RIPK3 levels were positively correlated with the UACR of DKD patients, suggesting that podocyte RIPK3 may influence the progression of DKD, which is consistent with the findings of podocyte‐specific RIPK3 KO in mice. GSK’872 is a specific inhibitor of RIPK3 activity that can suppress disease progression attributed to RIPK3 activation across multiple disease models.^[^ ^19^, ^20^ ^]^ In this study, GSK’872 alleviated podocyte damage, reduced proteinuria, and delayed progression of DKD in STZ/HFD/UNI and db/db mice. Overall, inhibition of RIPK3 presents a promising therapeutic strategy for DKD.

RIPK3 is a threonine/serine protein kinase initially believed to mediate necroptosis.^[^ ^11^, ^12^ ^]^ Upon activation, RIPK3 induces phosphorylation of MLKL, which promotes MLKL translocation to the plasma membrane, leading to programmed necroptosis characterized by calcium influx and plasma membrane damage.^[^ ^12^ ^]^ Emerging studies have demonstrated that RIPK3 can mediate biological effects beyond necroptosis. In a unilateral ureteral obstruction induced renal fibrosis model, RIPK3 promotes fibrotic progression via AKT‐dependent activation of ATP‐citrate lyase, independent of MLKL‐mediated necroptosis.^[^ ^21^ ^]^ Furthermore, in cardiac ischemia‐reperfusion injury models, RIPK3 exacerbates mitochondrial‐mediated apoptosis by suppressing FUNDC1‐dependent mitophagy.^[^ ^22^ ^]^ Although some studies have reported HG‐induced RIPK3/pMLKL activation and necroptosis in podocytes in vitro,^[^ ^13^, ^17^ ^]^ such pronounced morphological abnormalities of podocyte necrosis are not characteristic pathological features of DKD. Importantly, in a prior study, pMLKL staining was negative in the glomeruli of DKD patients.^[^ ^18^ ^]^ In this study, expression of RIPK3 and pRIPK3 was increased, while there were no significant changes to expression of MLKL and pMLKL in cultured podocytes treated with HG in vitro. The flow cytometry results revealed that necrosis, as determined by Annexin V^−^/propidium iodide^+^ staining, did not increase in response to HG intervention, while cell death increased. Furthermore, knockdown or inhibition of RIPK3 significantly restored podocyte marker proteins and reduced cell death induced by HG. However, knockdown of MLKL did not reverse the reduced expression of podocyte marker proteins and cell death, indicating that RIPK3, but not MLKL, was involved in HG‐induced podocyte injury. Besides, PAS staining and electron microscopy revealed no morphological evidence of podocyte necrosis in DKD mice, further indicating that RIPK3 promoted podocyte injury of DKD probably independent of necroptosis.

To further explore the mechanisms of RIPK3 to promote podocyte injury, RNA sequencing of mouse podocytes was conducted. GO and KEGG pathway enrichment analyses revealed significant enrichment in inflammation‐related pathways, with the NF‐κB signaling pathway being the most prominent, rather than necroptosis pathways. It is well established that inflammation is a major pathogenic mechanism in the development of DKD with activation of the NF‐κB p65 pathway clearly contributing to podocyte injury.^[^ ^7^, ^23^, ^24^ ^]^ Activation of NF‐κB p65 is characterized by its nuclear translocation. Previous studies have demonstrated that phosphorylation of NF‐κB p65 at Ser536 promotes its nuclear translocation, representing the active form of NF‐κB p65.^[^ ^25^ ^]^ In diabetic mice, both podocyte‐specific RIPK3 KO and pharmacological inhibition of RIPK3 activity significantly reduced the expression of NF‐κB downstream inflammatory factors. In vitro, RIPK3 knockdown attenuated podocyte injury, suppressed NF‐κB p65 nuclear translocation, and decreased inflammatory cytokine production, whereas RIPK3 overexpression exacerbated these pathological manifestations. Crucially, co‐immunoprecipitation assays confirmed an interaction between RIPK3 and NF‐κB p65 in vitro, with RIPK3 facilitating phosphorylation of NF‐κB p65 at Ser536. Furthermore, silencing of NF‐κB p65 alleviated podocyte injury induced by RIPK3 overexpression in the in vitro HG models, indicating that podocyte injury induced by RIPK3 involves activation of NF‐κB p65.

In conclusion, RIPK3 was upregulated in the glomerular podocytes of DKD patients and mice. Podocyte‐specific RIPK3 KO significantly alleviated podocyte injury and albuminuria in DKD mice. The RIPK3 kinase inhibitor GSK'872 replicated these renoprotective effects. Mechanistically, RIPK3 promoted podocyte injury through activation of the NF‐κB p65‐mediated inflammatory response without triggering necroptosis. Finally, the RIPK3‐NFκB p65 axis participates in regulating podocyte injury by inhibiting NF‐κB p65 in vitro. These results shed new light on the underlying pathogenic mechanisms of podocyte injury in DKD and highlight the therapeutic potential of mitigating podocyte injury through inhibition of the RIPK3/NF‐κB p65‐mediated inflammatory response as a viable treatment option for DKD (**Figure** **8**).

## 4 Experimental Section

### Human Renal Biopsy Specimens

The protocol of the human study was approved by the Research Ethics Committee of Guangdong Provincial People's Hospital (Ethics No. KY2024‐199‐02) and conducted in accordance with the tenets of the Declaration of Helsinki. Written informed consent was obtained from all subjects. Renal biopsy specimens were collected from DKD patients at the Department of Nephrology of Guangdong Provincial People's Hospital (Guangzhou, China). The inclusion criteria for DKD were as follows:(1) Age ≥ 18 years, (2) Established diagnosis of diabetes mellitus (based on standard diagnostic criteria), (3) Renal biopsy‐proven DKD, with exclusion of concurrent primary glomerulonephritis and other secondary glomerular diseases. Normal control samples were obtained from patients with renal tumors but not diabetes mellitus. All surgeries were performed at the Department of Urology of Guangdong Provincial People's Hospital. The clinical data are shown in Table S1 (Supporting Information).

### Animal Experimental Protocol

The protocol of the animal study was approved by the Research Ethics Committee of Guangdong Provincial People's Hospital (Ethics No. KY2024‐199‐02) and conducted in accordance with the “Guide for the Care and Use of Laboratory Animals”(https://grants.nih.gov/grants/olaw/guide‐for‐the‐care‐and‐use‐of‐laboratory‐animals.pdf).

Male C57BL/6, db/db, and wild‐type (db/m) mice (age, 6 weeks) were purchased from the Nanjing Biomedical Research Institute of Nanjing University (Nanjing, China) and housed in the Animal Centre of Guangzhou Forevergen Biosciences Co., Ltd. (Guangzhou, China) at a consistent room temperature of 25 °C ± 2 °C and relative humidity of 55% ± 5% under a 12‐h light/dark photoperiod to simulate a normal physiological environment with free access to standard rodent chow and water for a period of 16 weeks.

### Generation of Podocyte‐Specific RIPK3‐KO Mice

Ripk3^fl/fl^ mice (C57BL/6), generated by Shanghai Model Organisms Biotechnology Co., Ltd. (Shanghai, China), were crossed with Podocin‐Cre transgenic mice, which express Cre recombinase under control of the podocyte‐specific Podocin promoter, to produce Ripk3^fl/fl‐pod‐cre^ mice. Homozygous mice without the Podocin‐Cre transgene (Ripk3^fl/fl^) served as controls. Genomic DNA was extracted from the tails of 2‐week‐old mice for Flox genotyping, which was performed using specific primers (forward: GACACGGCACTCCTTGGTAT; reverse: CAGCGACACCTTGTGATC TCC). The mutant and wild‐type alleles produced fragments of 629 and 476 bp, respectively. Cre genotyping was performed using specific primers (forward: CGGTTATTCAACTTGCACCA; reverse: GCGCTGCTGCTCCAG). The Cre‐positive mice produce a fragment of 200 bp, while Cre‐negative mice produced no fragment.

### Construction of DKD Mouse Models

Two methods were used to construct mouse models of DKD. The first method involved traditional induction of diabetes using STZ combined with UNI and a HFD (60% of calories from fat, Research Diets, D12492) to promote glomerular lesions and increased proteinuria in the DKD model.^[^ ^8^ ^]^ Seven‐week‐old male C57BL/6, Ripk3^fl/fl^, and Ripk3^fl/fl‐pod‐cre^ mice underwent UNI, while mice in the control group underwent sham surgery. One‐week post‐UNI, mice in the surgical groups received low‐dose STZ injections (dissolved in 50 mmol L^−1^ sodium citrate buffer, pH 4.5) at a dosage of 50 mg kg^−1^ body weight per day for five consecutive days, while mice in the sham surgery group received injections of sodium citrate buffer. All surgical post‐UNI mice were fed an HFD for 17 weeks, while mice in the sham surgery group were fed a control diet (Research Diets, D12450J). The second method utilized spontaneously diabetic db/db mice, with their heterozygous littermates (db/m) serving as controls.

All mice had unrestricted access to food and water throughout the study. At the end of the experimental period, blood and urine samples were collected for biochemical analysis. Mice were then euthanized, and kidney samples were harvested for histopathological analysis. The cortical tissues of the kidneys were dissected for protein or RNA extraction for subsequent analysis.

### RIPK3 Kinase Inhibitor Intervention

To further explore new therapeutic strategies for DKD based on RIPK3 inhibition, the STZ/HFD/UNI and db/db mice, both at 16 weeks of age, were administered the RIPK3 kinase inhibitor GSK'872 (GSK; 1 mg kg^−1^) (Selleck Chemicals, Houston, TX, USA) via intraperitoneal injection every other day for 8 weeks.^[^ ^26^ ^]^ Control mice received an equal volume of dimethyl sulfoxide (DMSO). At the end of the experiment, blood and urine samples were collected for biochemical analysis. The mice were then euthanized and kidney samples were harvested for histopathological examination. The cortical kidney tissues were dissected for protein or RNA extraction for subsequent analysis.

### Blood and Urine Analysis

Mouse urinary albumin (Bethyl Laboratories Inc., TX, USA) and creatinine (Cayman Chemical, MI, USA) concentrations were measured using commercial kits in accordance with standard procedures.

### Renal Histologic Analysis

Mouse kidneys were fixed with 4% paraformaldehyde for 24 h at 4 °C, embedded in paraffin, and cut into 4 µm‐thick sections, which were stained with PAS stain (Solarbio, Beijing, China).

### Cell Culture and Treatment

Conditionally immortalized mouse podocytes were kindly provided by Dr. Jochen Reiser (Rush University Medical Center, Chicago, IL) and cultured as previously described.^[^ ^7^ ^]^ To simulate podocyte injury in DKD in vitro, cultured podocytes were treated with an HG concentration of 30 mM for 72 h, whereas normal glucose (CON, 5.3 mM) was used as a control and mannitol (MA, 24.7 mM) as an osmotic control.^[^ ^7^ ^]^ Two interventions were employed for knockdown and inhibition of podocyte RIPK3: (1) RIPK3‐targeting siRNA (si ‐RIPK3) to knock down podocyte RIPK3 expression, followed by HG treatment for 72 h, to produce the experimental groups CON, MA, HG, HG+siRNA negative control (HG+NC), and HG+si‐RIPK3; and (2) HG‐treated podocytes were cultured with or without RIPK3 kinase inhibitor GSK'872(GSK) at varying concentrations (0.1, 0.3, and 1 µM) for 72 h to produce the groups CON+DMSO (vehicle control)(CON), MA+DMSO(MA), HG+DMSO(HG), and HG+GSK (0.1, 0.3, and 1 µM). Podocytes were transfected with a lentivirus (HanBio Technology Co. Ltd, Shanghai, China) overexpressing RIPK3 (oe‐RIPK3) or a control lentivirus (Vector) to produce the groups CON+Vector, MA+Vector, HG+Vector, and HG+oe‐RIPK3. For knockdown and inhibition of podocyte NF‐κB p65, siRNA targeting NF‐κB p65 and JSH‐23 (20 µM, an inhibitor of NF‐κB transcriptional activity) (Selleck Chemicals, Houston, TX, USA) were used to produce the experimental groups CON, MA, HG, HG+DMSO, HG+NC, HG+JSH‐23, and HG+si‐p65. To activate podocyte NF‐κB p65, podocytes were treated with NF‐κB activator 1 (AC1; 1, 2, 3 µM) (Selleck Chemicals, Houston, TX, USA) for 72 h. To investigate the functional relationship between RIPK3 and the NF‐κB p65 pathway, a rescue experiment was conducted with HG‐induced podocyte injury models by knockdown and overexpression of RIPK3, followed by the addition of AC1 (3 µM) and NF‐κB p65‐targeting siRNA, to produce two sets of experimental groups: (1) CON+Vector+NC, HG+Vector+NC, HG+oe‐RIPK3+NC, HG+si‐P65+Vector, and HG+oe‐RIPK3+si‐P65; and (2) CON, HG, HG+DMSO, HG+siRIPK3, HG+AC1, HG+AC1+NC, and HG+AC1+siRIPK3.

### siRNA Transfection

The siRNAs targeting mouse MLKL, RIPK3, and NF‐κB p65, and a negative control, were synthesized by RiboBio Co., Ltd. (Guangzhou, China). Podocytes were transfected with siRNA (50 nmol L^−1^) for 6 h using Lipofectamine 2000 transfection reagent (Thermo Fisher Scientific, Waltham, MA, USA), followed by HG medium replaced for 72 h.

### Western Blot

Total proteins from mouse kidney cortex and podocytes were extracted using RIPA lysis buffer (Beyotime Institute of Biotechnology, Jiangsu, China) containing phenylmethylsulfonyl fluoride (Beyotime Institute of Biotechnology, Jiangsu, China). Nuclear proteins from podocytes were extracted using a commercial nuclear and cytoplasmic protein extraction kit (Beyotime Institute of Biotechnology, Jiangsu, China). The protein concentrations were measured using a bicinchoninic acid protein assay kit (Thermo Fisher Scientific, Waltham, MA, USA). Following denaturation with loading buffer (Takara, Japan), the proteins were separated by electrophoresis using 4%–20% gradient gels and then electroblotted onto polyvinylidene difluoride membranes(Millipore, MA, USA), which were incubated overnight at 4 °C with primary antibodies against GAPDH (60004‐1‐Ig, dilution 1:5000; Proteintech, Wuhan, China), RIPK3 (ab62344, dilution 1:1000; Abcam, Cambridge, MA, USA), phosphorylated (p)‐RIPK3 (S232) (ab195117, dilution 1:1000; Abcam, Cambridge, MA, USA), MLKL (ab243142, dilution 1:1000; Abcam, Cambridge, MA, USA), p‐MLKL (S345) (ab196436, dilution 1:1000; Abcam, Cambridge, MA, USA), NF‐κB p65 (8242, dilution 1:1000; Cell Signalling Technology, MA, USA), p‐NF‐κB p65 (S536) (3033, dilution 1:1000; Cell Signalling Technology, MA, USA), Podocin (ab50339, dilution 1:1000; Abcam, Cambridge, MA, USA), Nephrin (ABT331, dilution 1:1000; Millipore, MA, USA), and H3 (4499, dilution 1:1000; Cell Signalling Technology, MA, USA), followed by secondary horseradish peroxidase‑conjugated goat anti‑rabbit (7074, dilution 1:5000; Cell Signalling Technology, MA, USA) or anti‐mouse (7076, dilution 1:5000; Cell Signalling Technology, MA, USA) secondary antibodies for 1 h at room temperature. Finally, the protein bands were detected using Pierce™ ECL Western Blotting Substrate (Thermo Fisher Scientific, Waltham, MA, USA) and visualized with an ImageQuant^TM^ LAS500 instrument (GE Healthcare Life Sciences, USA).

### RT‐qPCR

Total RNA from renal tissues or cultured podocytes was extracted with TRIzol™ reagent (Thermo Fisher Scientific, Waltham, MA, USA) and reverse‐transcribed into cDNA using the PrimeScript™ RT Reagent Kit (Takara Biotechnology, Dalian, China). The cDNA was amplified by RT‐qPCR using Power SYBR™ Green PCR Master Mix (Takara Biotechnology, Dalian, China) and the primers listed in Table S2 (Supporting Information). The mRNA expression levels were quantified using the 2^−ΔΔCq^ method against GAPDH as an internal control.

### Immunofluorescence

Paraffin‐embedded sections were dewaxed and rehydrated, followed by epitope retrieval. Paraffin‐embedded sections and podocytes were fixed with 4% paraformaldehyde for 10 min at room temperature, permeabilized with 0.1% Triton X‐100 for 10 min at room temperature, blocked with 5% bovine serum albumin (BSA), and incubated overnight at 4 °C with primary antibodies against RIPK3 (17563‐1‐AP, dilution 1:200; Proteintech, Wuhan, China) and synaptopodin (sc‐515842, dilution 1:200; Santa Cruz Biotechnology, CA, USA), followed by goat anti‐mouse Alexa Fluor 488 (4408, dilution 1:500; Cell Signalling Technology, MA, USA) and goat anti‐rabbit Alexa Fluor 555 (4413, dilution 1:500; Cell Signalling Technology, MA, USA) fluorescent secondary antibodies at room temperature for 1 h. The nuclei were stained with 4′,6‐diamidino‐2‐phenylindole. Finally, the sections and podocytes were imaged under a confocal microscope (LSM900, Zeiss). Co‐localized synaptopodin and RIPK3 immunofluorescence signals (appearing as yellow overlapping areas) of each image of the kidney sections were analyzed using Image J software. The percentage of glomerular podocyte RIPK3‐positive area was calculated by dividing the measured area of positive signals by the corresponding glomerular cross‐sectional area.

### Immunohistochemical Analysis

Paraffin‐embedded sections were dewaxed and rehydrated, followed by epitope retrieval, then fixed with 4% paraformaldehyde for 10 min at room temperature, permeabilized with 0.1% Triton X‐100 for 10 min at room temperature, and blocked with 5% BSA for 1 h at room temperature. The sections and cells were incubated overnight at 4 °C with primary antibodies against TNF‐α (ab6671, dilution 1:200; Abcam, MA, USA) or WT1(ab89901, dilution 1:200; Abcam, MA, USA). After washing three times with phosphate‐buffered saline (PBS), the sections were incubated with horseradish peroxidase‐conjugated goat anti‐rabbit secondary antibodies for 30 min at 37 °C and then stained with 3,3′‐diaminobenzidine and hematoxylin to label the nuclei. The positively stained areas of each image of the kidney sections were analyzed using Image‐Pro Plus software (Media Cybernetics, Inc., Rockville, MD, USA).

### Flow Cytometry

Podocyte death was analyzed using a commercial apoptosis detection kit (KeyGEN BioTECH, Jiangsu, China). Briefly, podocytes were washed three times with ice‐cold PBS, digested with 0.25% ethylenediaminetetraacetic acid‐free trypsin, collected, washed twice with PBS, and resuspended in 500 µL of binding buffer. Then, 5 µL each of fluorescein isothiocyante‐conjugated Annexin V and propidium iodide were added and the suspended cells were incubated at room temperature for 15 min. Afterward, fluorescence was detected using a BD FACSVerse™ flow cytometer (BD Biosciences, Franklin Lakes, NJ, USA).

### Transmission Electron Microscopy

Kidney tissue specimens were examined using an electron microscope (H‐700, Hitachi, Tokyo, Japan) as previously reported.^[^ ^7^ ^]^

### RNA‐Sequencing Analysis

Total RNA was extracted from cultured podocytes using TRIzol™ reagent. Library construction and sequencing were carried out as previously reported.^[^ ^27^ ^]^ Quality control of the raw sequencing data was performed using the fastp tool (https://github.com/OpenGene/fastp). The cleaned sequencing reads were then aligned to the human reference genome using the HISAT2 program (https://daehwankimlab.github.io/hisat2/). Gene and transcript assembly was conducted with the StringTie assembler (https://github.com/gpertea/stringtie). Expression levels are reported as FPKM values (Fragments Per Kilobase of transcript per Million mapped reads). Differentially expressed genes between samples were analyzed using the R package “edgeR” (https://bioconductor.org/ packages/release/bioc/html/edgeR.html), with significance thresholds of fold change > 2 or < 0.5 and a probability (*p*) value < 0.05. Finally, functional enrichment analysis of the differentially expressed genes was conducted using The Database for Annotation, Visualization, and Integrated Discovery (DAVID) (https://davidbioinformatics.nih.gov/) for GO and KEGG pathway analysis.

### Data Analysis

The data are presented as the mean ± standard deviation (SD). Data analysis was performed using IBM SPSS Statistics for Windows (version 23.0; IBM Corporation, Armonk, NY, USA) and the results were plotted using Prism 9 software (GraphPad Software, LLC, San Diego, CA, USA). The two‐sided Student's *t*‐test and one‐way analysis of variance were used to compare differences between and among groups, respectively. Fisher's least significant difference test was used for multiple comparisons with homogeneous variance and Dunnett's T3 method was used for multiple comparisons with heterogeneous variance. Correlations between the two variables were analyzed using Spearman's rho correlation coefficients. A *p*‐value < 0.05 was considered statistically significant.

## Conflict of Interest

The authors declare no conflict of interest.

## Author Contributions

L.L.A. planned and performed experiments, analyzed data, and drafted the manuscript. L.J.Y., L.R.Z., Z.X.C., and C.Y.H. helped to perform the animal and cell experiments. C.Y.T., Y.Y., W.W.T., and Z.S.Q. helped to analyze the data and revise and edit the manuscript. Z.L and L.X.L., the principal investigators, conceived the scientific ideas, oversaw the project, designed the experiments, and refined the manuscript. All authors have read and approved the article.

## Supporting information



Supporting Information

## Data Availability

All data supporting the findings of this study are available within the article and its supporting information files and from the corresponding author upon reasonable request.
