# Supplementary material for: Podocyte RIPK3 Deletion Improves Diabetic Kidney Disease by Attenuating NF‐κB p65 Driven Inflammation
Source: Adv Sci (Weinh). 2025 Jun 20;12(33):e03325. doi: 10.1002/advs.202503325 (PMC12412576; doi:10.1002/advs.202503325)
Supplement: Supplementary file 1 — Supporting Information [file ADVS-12-e03325-s001.docx]

**Supporting Information for**

**“Podocyte RIPK3 deletion improves diabetic kidney disease by attenuating NF-κB p65 driven inflammation”**

Lu’an Li ^1^, Jiaying Li ^1^, Ruizhao Li ^1^, Xingchen Zhao ^1^, Yuanhan Chen ^1^, Yating Cai ^1^, Yan Yang ^1^, Weiteng Wang ^1^, Siqi Zheng ^1^, Li Zhang ^1, *^, Xinling Liang ^1, *^

^1^ Department of Nephrology, Guangdong Provincial People's Hospital (Guangdong Academy of Medical Sciences), Southern Medical University, Guangzhou, 510080, China.

^*^ Correspondence:

Xinling Liang or Li Zhang, Department of Nephrology, Guangdong Provincial People’s Hospital (Guangdong Academy of Medical Sciences), Southern Medical University. No.106, Zhongshan Second Road, Yuexiu district, Guangzhou, 510080, Guangdong, China.

E-mail: liangxinling@gdph.org.cn or zhangli7595@gdph.org.cn

**Supplemental methods**

**Co-immunoprecipitation**

Podocytes were harvested and extracted in IP lysis buffer (Beyotime Institute of Biotechnology, Jiangsu, China) containing phenylmethylsulfonyl fluoride (Beyotime Institute of Biotechnology, Jiangsu, China). Equal amounts of proteins were incubated with mouse anti-RIPK3(sc-374639, dilution 1:200; Santa Cruz Biotechnology, CA, USA) or rabbit anti-NF-κB p65 (8242, Cell Signalling Technology, MA, USA) antibody at 4°C overnight, and then the Dynabeads Protein G (Thermo Fisher Scientific, Waltham, MA, USA) were added and incubated for 2h. Finally, proteins were separated from Dynabeads Protein G and detected by Western blot.

**Luciferase reporter assays**

HEK293T cells were transfected with a mixture of 200 ng of pGL3-NF-κB luciferase reporter (firefly luciferase) and 20 ng of pRL-TK (Renilla luciferase plasmid), together with NF-κB p65 expression plasmid or empty vector plasmid. Furthermore, the HEK293T cells were treated with GSK’872 (0.3μM) for 72 h. Luciferase activity was measured by using a Dual-Luciferase Reporter Assay System kit (Promega, San Luis Obispo, CA) according to the manufacturer’s protocol. Data represent relative firefly luciferase activity, normalized to Renilla luciferase activity.


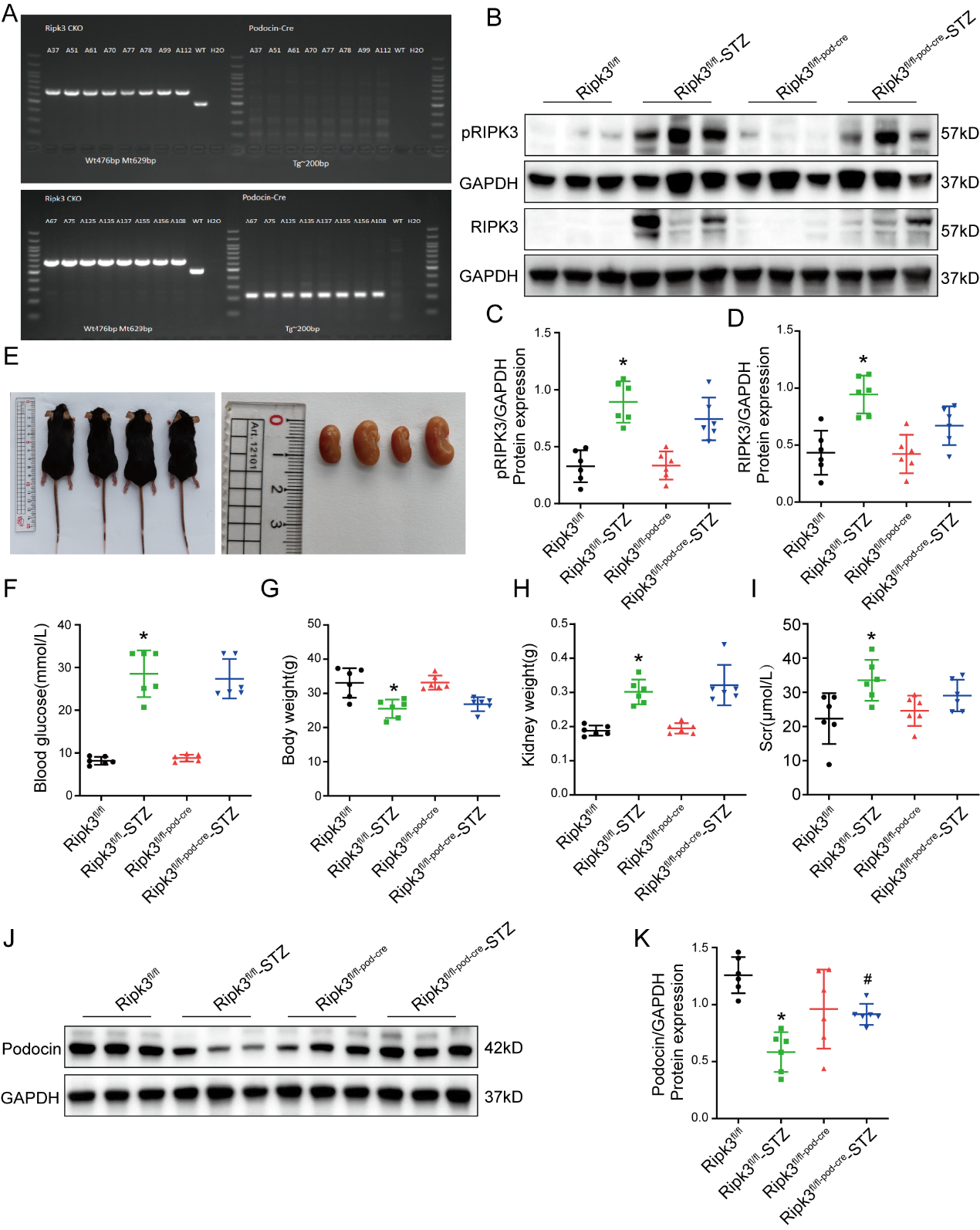


**Figure S1. Baseline data of** **podocyte-specific RIPK3-KO mice**

(A) Representative agarose gel electrophoresis images to verify genotyping in podocyte-specific RIPK3-KO mice. (B-D) Representative western blot images and quantification of RIPK3 and pRIPK3 in the renal cortex of mice (n = 6). (E) Representative mice and kidney images. (F) Blood glucose in mice (n = 6). (G-H) Quantification of body weight and kidney weight in mice (n = 6). (I) Serum creatinine (Scr, n = 6) in mice. (J-K) Representative western blot image and quantification of podocin in the renal cortex of mice (n = 6). Data are shown as the mean ± SD. * vs. Ripk3^fl/fl^, ^#^ vs. Ripk3^fl/fl^-STZ, *p* < 0.05.


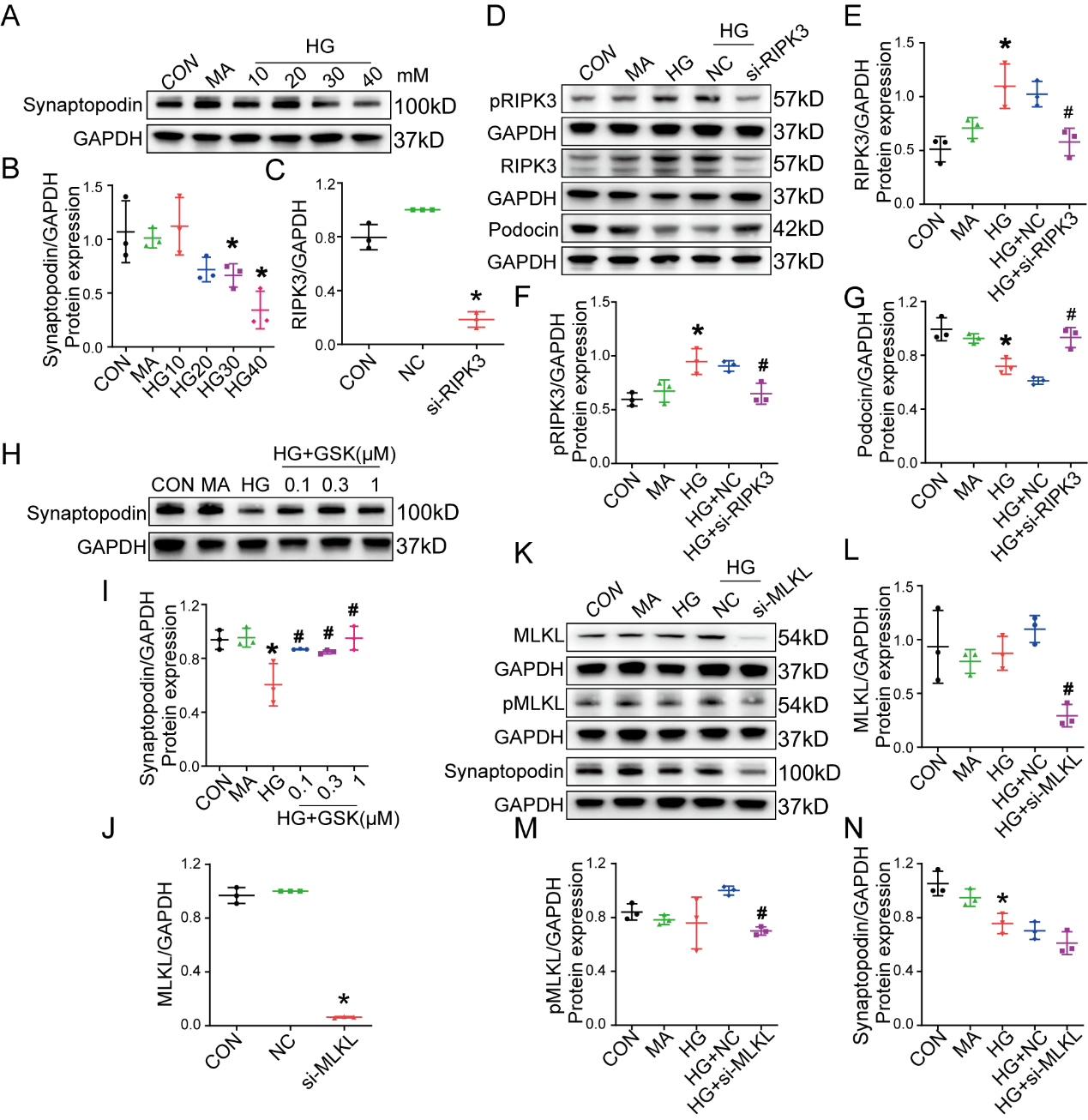


**Figure S2. RIPK3, but not MLKL, was involved in HG-induced podocyte injury**

(A-B) Representative western blot images and quantification of synaptopodin (n = 3) in podocytes treated with varying concentrations of high glucose (HG) (10, 20, 30, and 40 mM) for 72 h. (C) Relative mRNA level of RIPK3 (n = 3) in podocytes treated with RIPK3-targeting siRNA. (D-G) Representative western blot image and quantification of RIPK3, pRIPK3 and podocin in podocytes treated with RIPK3-targeting siRNA (n = 3). (H-I) Representative western blot image and quantification of synaptopodin (n = 3) in podocytes treated with GSK’872(GSK). (J) Relative mRNA level of MLKL in podocytes treated with MLKL-targeting siRNA (n = 3). (K-N) Representative Western blot image and quantification of MLKL, pMLKL and synaptopodinin podocytes treated with MLKL-targeting siRNA (n = 3). Data are shown as the mean ± SD. * vs. NC and MA, ^#^ vs. HG and HG+NC, *p* < 0.05.


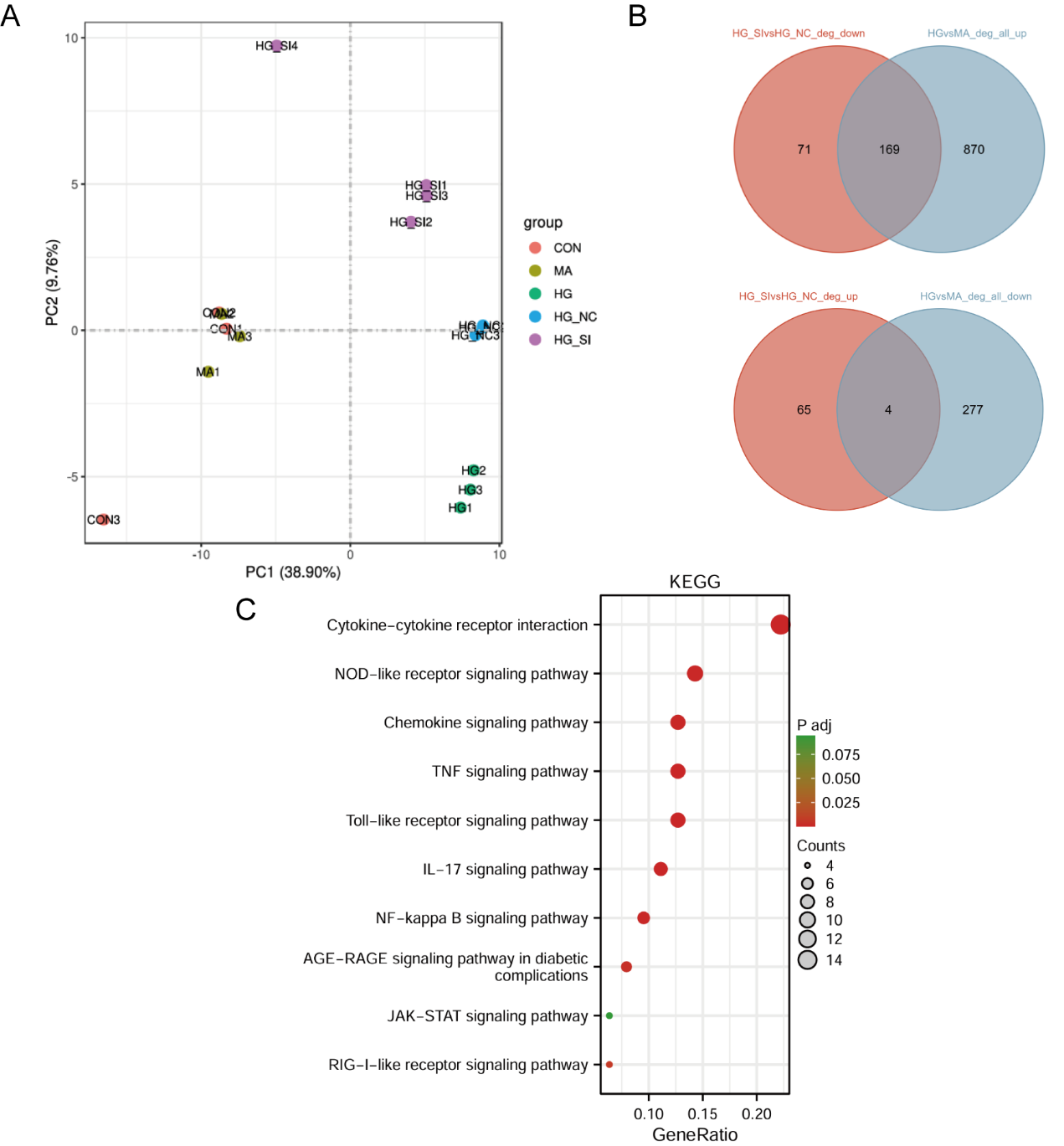


**Figure S3. RNA-sequencing Analysis**

(A) PCA analysis. (B) Venn diagram. (C) KEGG pathway analysis.


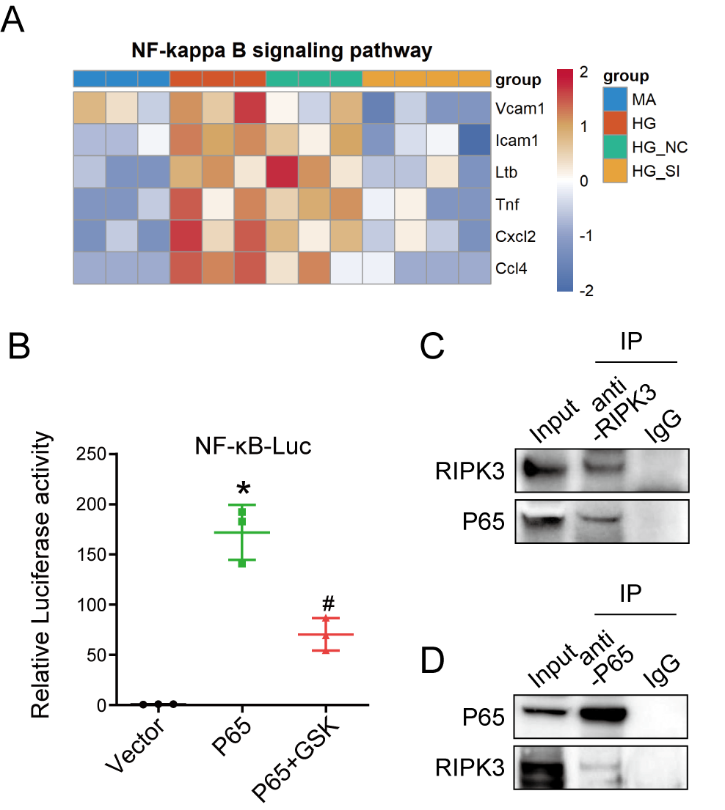


**Figure S4. RIPK3 promoted activation of the NF-κB p65 inflammatory pathway**

(A) Genes enriched in the NF-κB signaling pathway. (B) HEK293T cells were co-transfected with pGL3-NF-κB luciferase reporter (NF-κB-Luc), pRL-TK, and NF-κB p65 expression plasmid or vector plasmid. Furthermore, the HEK293T cells were treated with GSK’872 (GSK) for 72 h. Cell lysates were harvested, NF-κB-Luc reporter activity was determined by dual luciferase reporter assays. (C-D) Total proteins of podocytes were incubated with anti-RIPK3 or anti-NF-κB p65. Precipitated and unprecipitated protein fractions were analyzed by Western blot using anti-RIPK3 antibody or anti-NF-κB p65 antibody (n = 3). Data are shown as the mean ± SD. * vs. Vector, # vs. P65, *p* < 0.05.


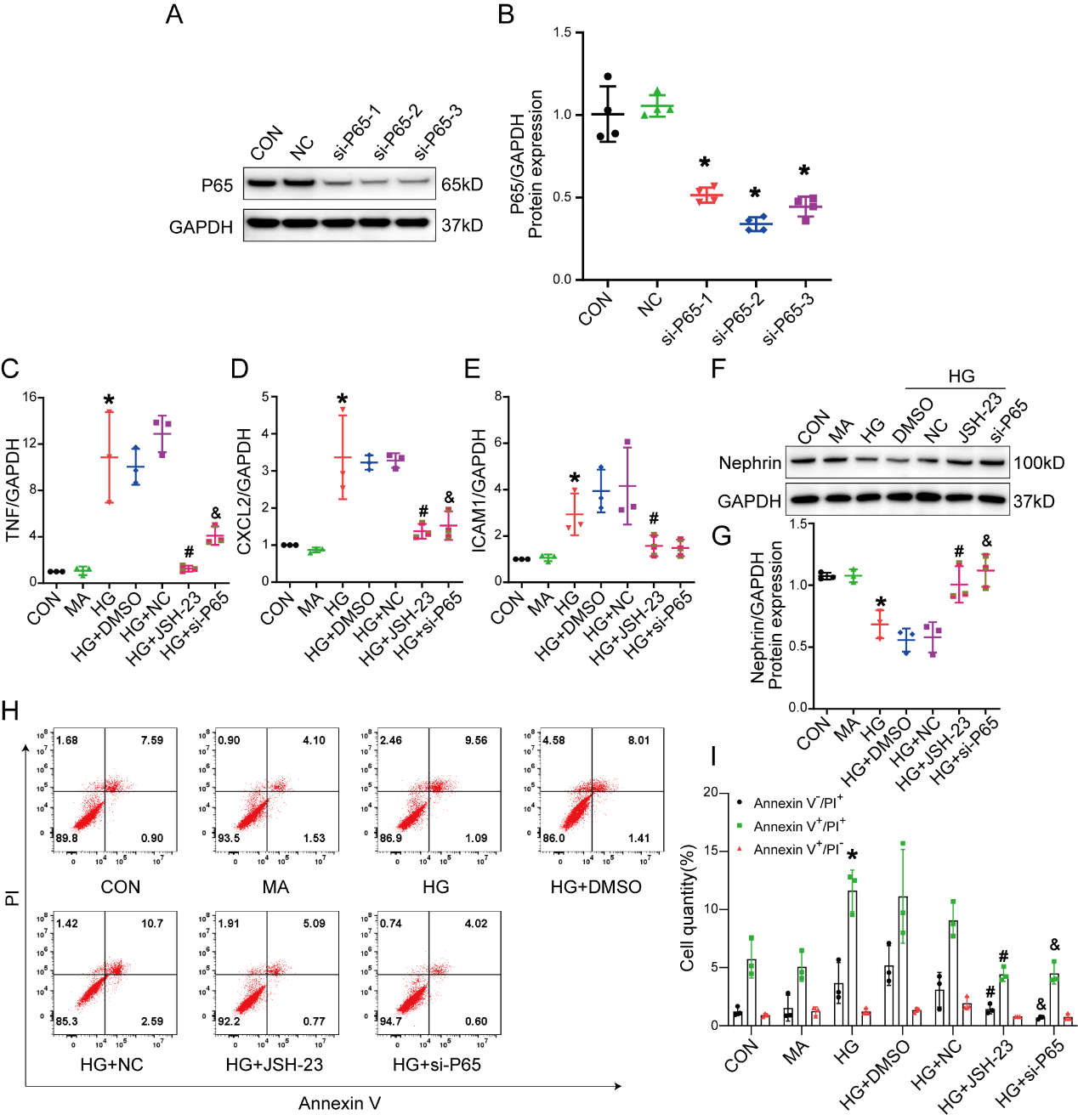


**Figure S5. Activation of the NF-κB p65 pathway contributed to HG induced podocyte injury**

(A-B) Representative western blot image and quantification of p65 (n = 3) in podocytes treated with P65-targeting siRNA. (C-E) Relative mRNA levels of TNF, CXCL2 and ICAM1 in podocytes treated with P65-targeting siRNA and JSH-23 (n = 3). (F-G) Representative western blot image and quantification of nephrin (n = 3) in podocytes treated with P65-targeting siRNA and JSH-23. (H-I) Flow cytometry analysis to assess the regulation of podocyte death by inhibiting p65 (n = 3). Data are shown as the mean ± SD. * vs. MA and NC, ^#^ vs. HG+DMSO, ^&^ vs. HG+NC, *p* < 0.05.


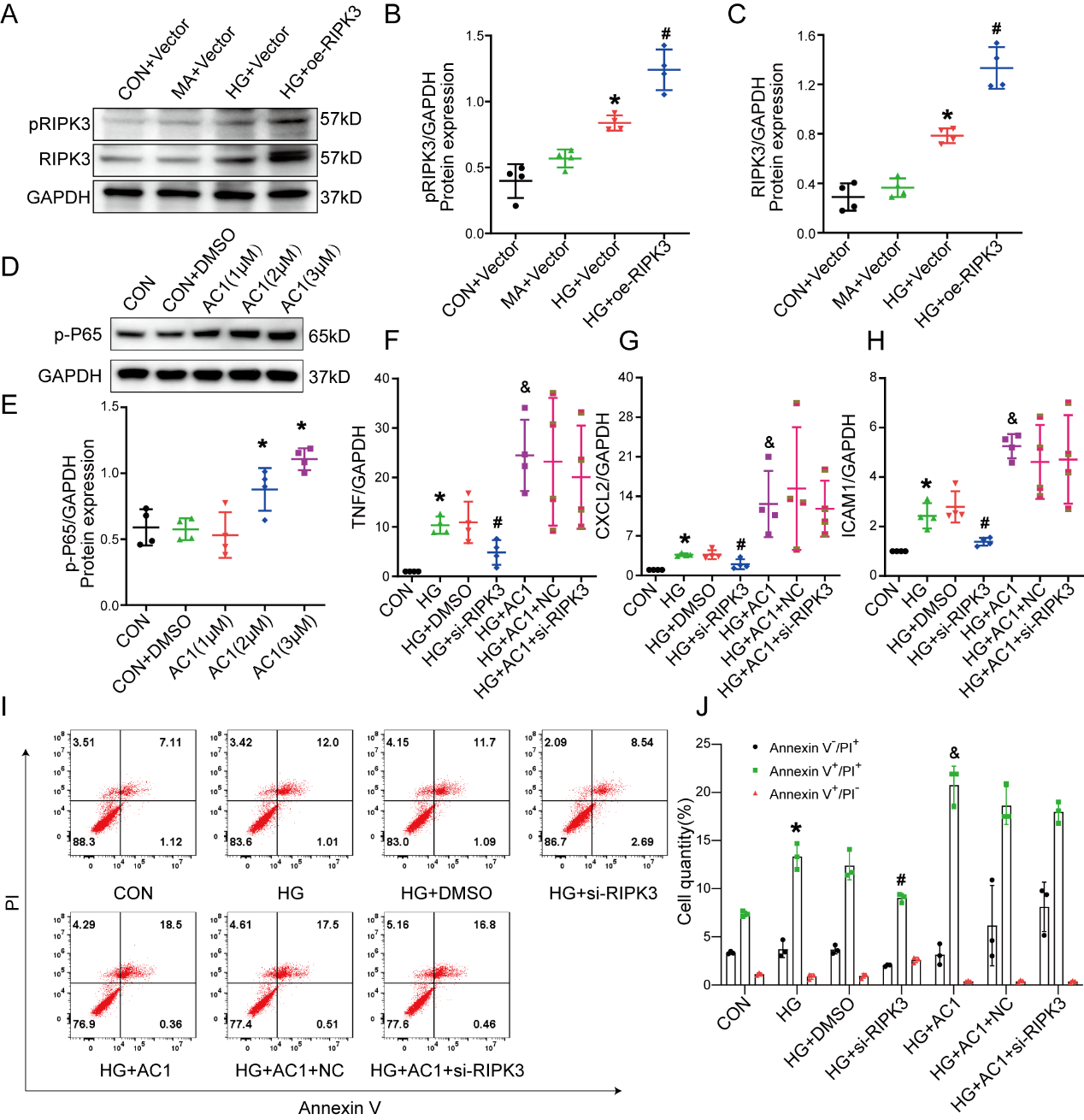


**Figure S6. RIPK3 promoted podocyte injury through the activation of the NF-κB P65 pathway**

(A-C) Representative western blot images and quantification of RIPK3 and pRIPK3 in podocytes (n = 4). (D-E) Representative western blot images and quantification of p-p65 (n = 3) in podocytes treated with varying concentrations of NF-κB activator 1 (10, 20, 30, and 40 mM). (F-H) Relative mRNA levels of TNF, CXCL2 and ICAM1 in podocytes (n = 4). (I-J) Flow cytometry analysis to assess the regulation of podocyte death (n = 3). Data are shown as the mean ± SD. * vs. CON, CON+DMSO and MA+Vector, ^#^ vs. HG and HG+Vector, ^&^ vs. HG+DMSO, *p* < 0.05.


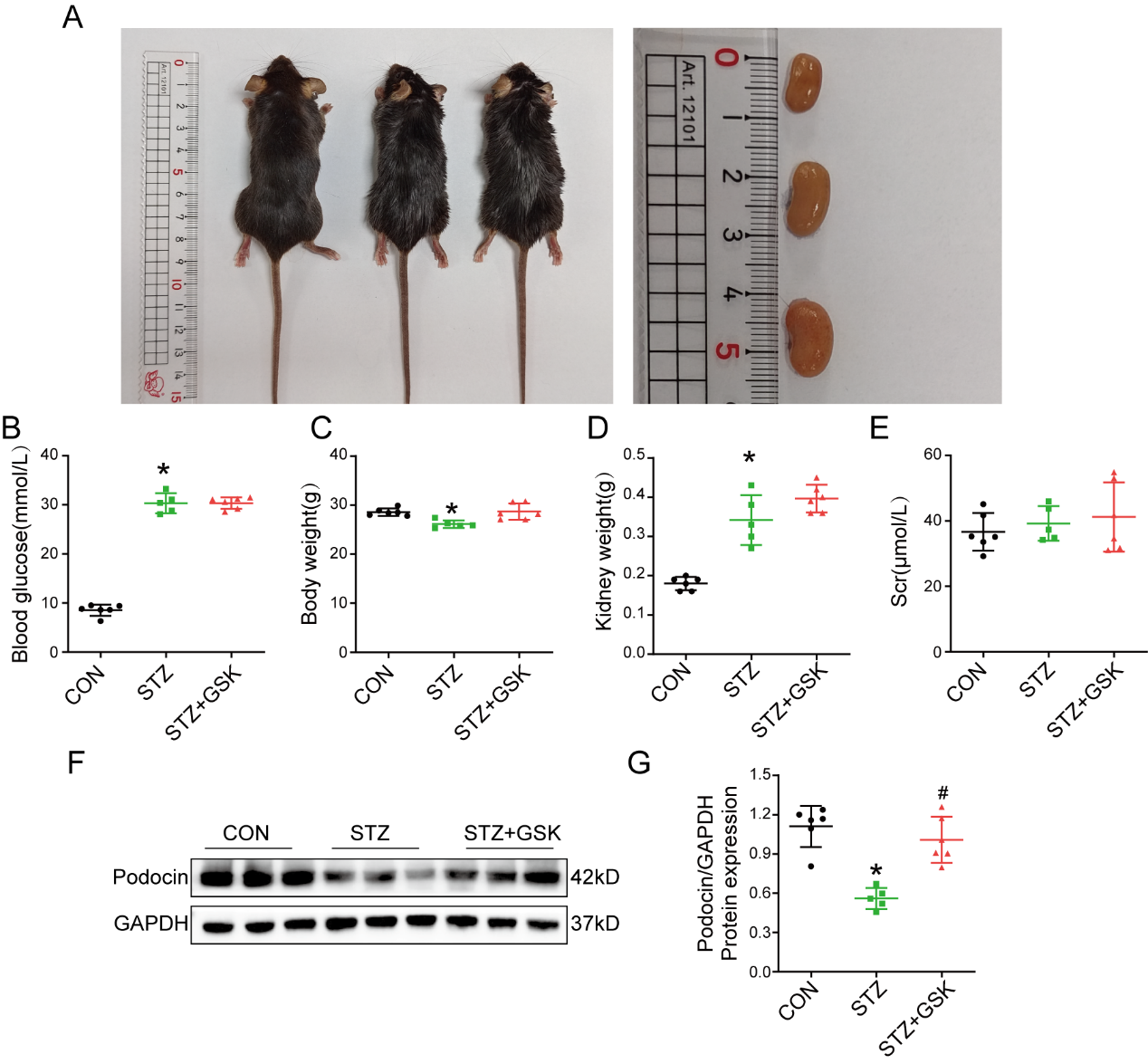


**Figure S7. Baseline data of STZ/HFD/UNI mice treated with GSK'872**

(A) Representative mice and kidney images. (B) Blood glucose in mice (n = 5-6). (C-D) Quantification of body weight and kidney weight in mice (n = 5-6). (E) Serum creatinine (Scr, n = 5-6) in mice. (F-G) Representative western blot image and quantification of podocin (n = 5-6) in the renal cortex of mice. Data are shown as the mean ± SD. * vs. CON, ^#^ vs. STZ, *p* < 0.05.


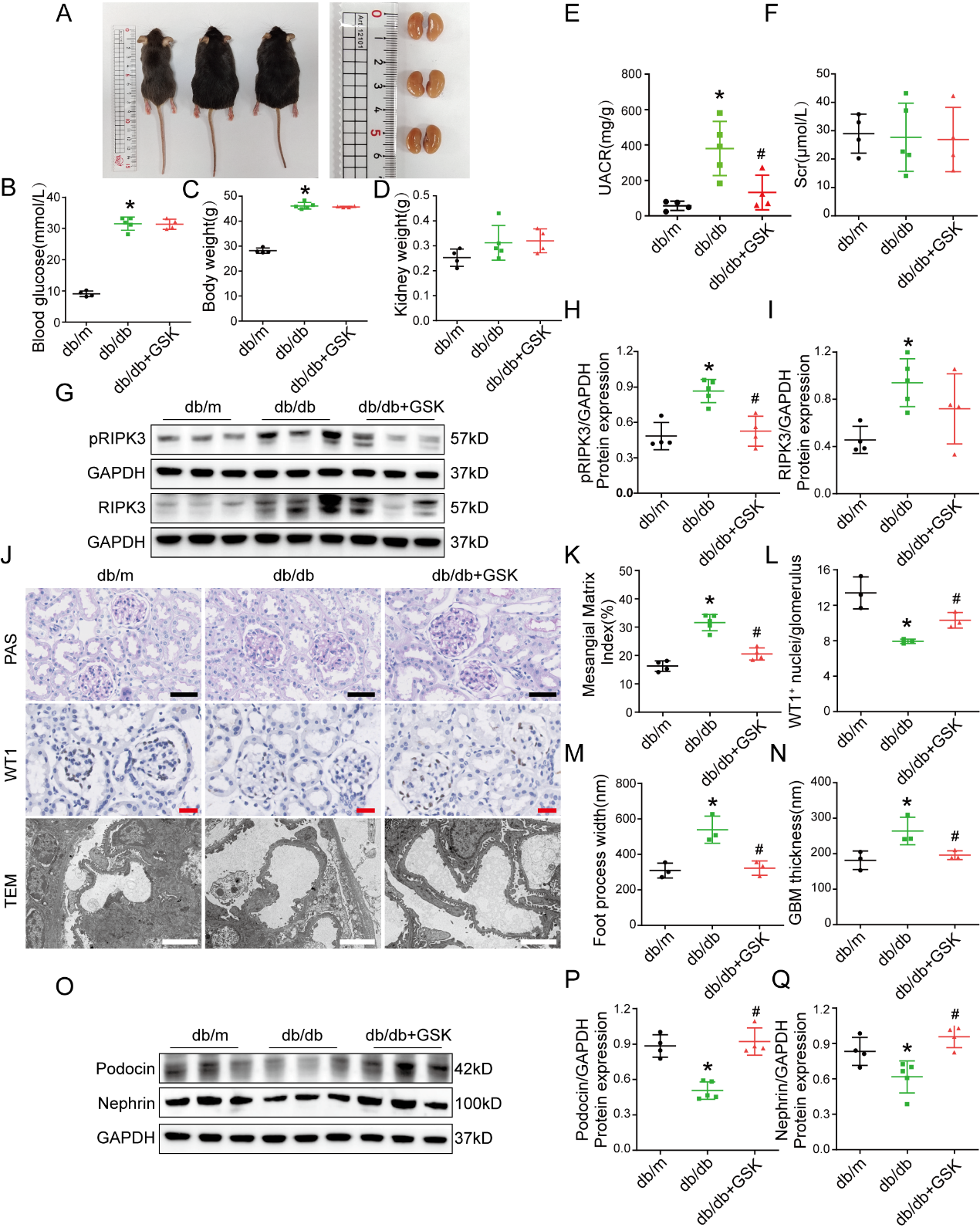


**Figure S8: GSK’872 protected against DKD in db/db mice**

(A) Representative mice and kidney images. (B) Blood glucose (n = 4-5) in mice. (C-D) Quantification of body weight and kidney weight in mice (n = 4-5). (E) UACR of mice (n = 4-5). (F) Scr of mice (n = 4-5). (G-I) Representative western blot image and quantification of pRIPK3 and RIPK3 in the renal cortex of mice (n = 4-5). (J) Representative PAS staining, TEM and WT1–stained images of glomeruli in kidney sections of mice. Scale bars: black 50μm, red 25μm, white 2μm. (K-N) Quantification of glomerular mesangial matrix expansion (n = 4-5), foot process width (n = 3), GBM thickness (n = 3) and WT1-positive cells (n = 3) in mice. (O-Q) Representative western blot image and quantification of nephrin and podocin in the renal cortex of mice (n = 4-5). Data are shown as the mean ± SD. * vs. db/m, ^#^ vs. db/db, *p* < 0.05.


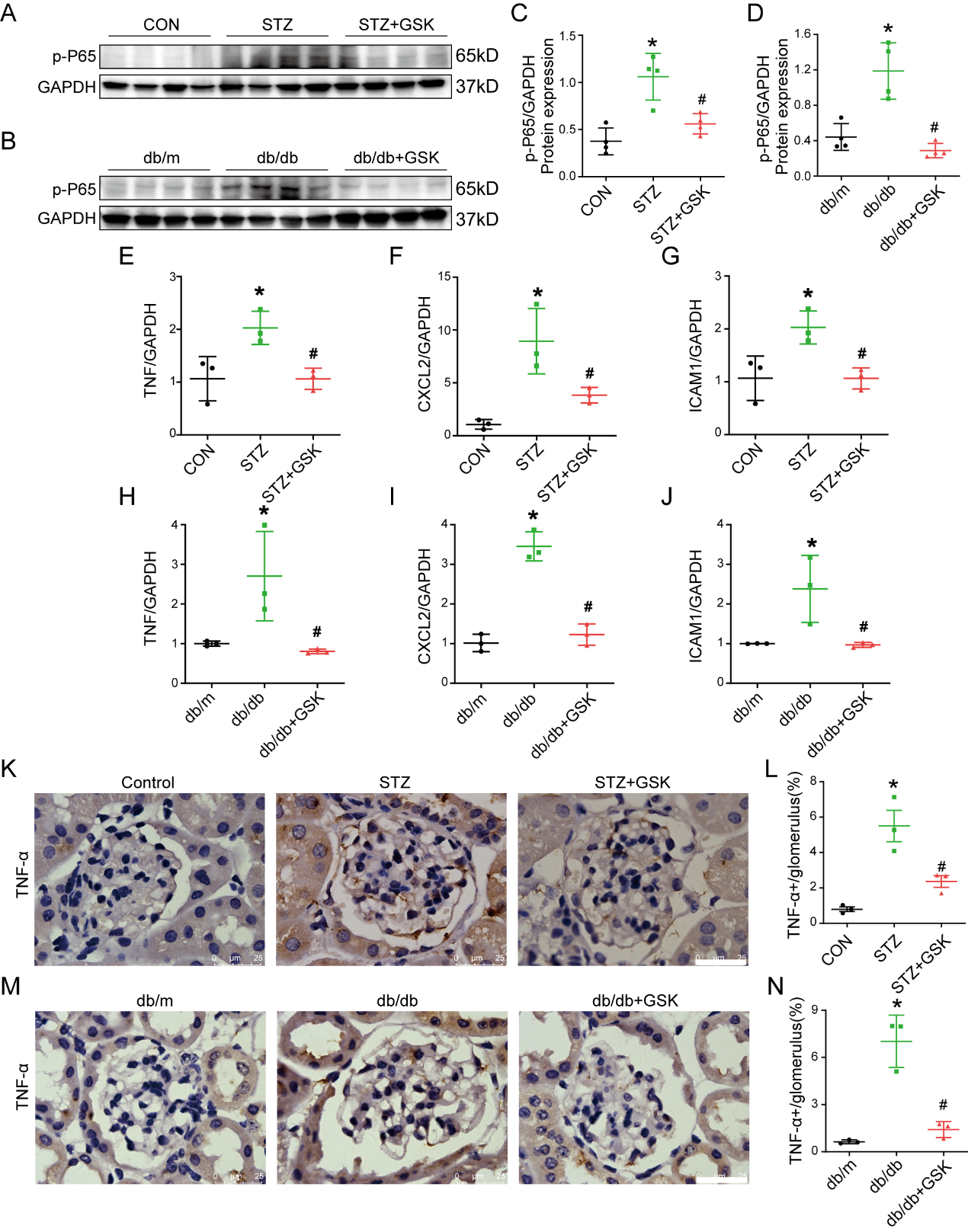


**Figure S9. GSK’872 suppresses the activation of the NF-κB p65 inflammatory pathway in DKD mice.**

(A-D) Representative western blot image and quantification of pNF-κB p65(p-P65) in the renal cortex of mice (n = 4). (E-J) Relative mRNA levels of TNF, CXCL2 and ICAM1 in the renal cortex of mice (n = 3). (K-N) Representative immunohistochemistry image and quantification of TNF-α in kidney sections of mice (n = 3). Scale bars: white 25μm. Data are shown as the mean ± SD. * vs. db/m and CON, ^#^ vs. db/db and STZ, *p* < 0.05.

**Table S1. The clinical parameters of patients performed with immunofluorescence**

| Control Group(N=7) | | | | | | | |
| --- | --- | --- | --- | --- | --- | --- | --- |
|  | RIPK3 positive area of podocytes/glomeruli (‰) | **Sex** | Age | Body weight（kg） | UACR（mg/gCr） | Scr（umol/L） | eGFR  （ml/min*1.73m2） |
| 1 | 0.85 | M | 54 | 72 | NA | 136.39 | 50.30 |
| 2 | 1.78 | F | 48 | 56.50 | NA | 108 | 52.30 |
| 3 | 0.71 | M | 65 | 64 | NA | 166.08 | 36.70 |
| 4 | 1.25 | F | 64 | 55 | NA | 77.71 | 65.50 |
| 5 | 2.25 | M | 48 | 66 | NA | 130.66 | 55.20 |
| 6 | 0.58 | M | 74 | 59 | NA | 126.67 | 47.80 |
| 7 | 0.61 | M | 79 | 55 | NA | 117.45 | 50.50 |
| DKD group(N=23) | | | | | | | |
| 1 | 15.92 | M | 42 | 64 | 2691.42 | 473.18 | 12.20 |
| 2 | 17.96 | M | 41 | 54.50 | 3126.85 | 123.94 | 61.86 |
| 3 | 14.94 | M | 75 | 55 | 2095.46 | 253.60 | 20.50 |
| 4 | 5.10 | M | 57 | 57 | 1604.65 | 276.46 | 20.96 |
| 5 | 8.24 | M | 53 | 61.60 | 822.13 | 84.65 | 90.20 |
| 6 | 9.38 | F | 48 | 51.80 | 5080.50 | 404.90 | 10.60 |
| 7 | 13.09 | M | 49 | 65 | 5538.98 | 266 | 23.20 |
| 8 | 3.21 | F | 31 | 56.10 | 385.67 | 407.55 | 11.86 |
| 9 | 0.14 | M | 74 | 64.60 | 1659.91 | 215.72 | 25.10 |
| 10 | 10.25 | M | 63 | 73.50 | 4947.30 | 236.69 | 27.00 |
| 11 | 8.44 | F | 59 | 44 | 8923.61 | 84.42 | 72.40 |
| 12 | 10.04 | F | 42 | 50.38 | 4826.81 | 104.78 | 56.50 |
| 13 | 8.27 | M | 52 | 70.01 | 3945.33 | 142.16 | 48.50 |
| 14 | 3.89 | M | 67 | 52.00 | 2336.07 | 114.89 | 72.18 |
| 15 | 10.23 | M | 58 | 49.20 | 3445.29 | 329.93 | 16.80 |
| 16 | 8.62 | M | 58 | 65 | 3541.41 | 295.7 | 19.20 |
| 17 | 8.06 | M | 51 | 57 | 3324.08 | 735 | 6.70 |
| 18 | 3.38 | F | 63 | 58 | 358.17 | 110.04 | 46.12 |
| 19 | 9.62 | M | 37 | 61.04 | 3953.36 | 277.24 | 24.06 |
| 20 | 4.67 | M | 45 | 72.30 | 1420.35 | 205.81 | 32.60 |
| 21 | 11.41 | M | 71 | 67 | 4264.96 | 258.88 | 25.88 |
| 22 | 8.32 | M | 65 | 57 | 97.83 | 242.70 | 23.20 |
| 23 | 7.87 | M | 50 | 66 | 4176.71 | 637.38 | 8 |

Scr, serum creatinine; UACR, urinary albumin creatinine ratio; eGFR, estimated glomerular filtration rate.

**Table S2. Sequences of primers for RT‒qPCR**

| GENE | Primer sequences |
| --- | --- |
| GAPDH | Forward:5’-AGGTCGGTGTGAACGGATTTG-3’  Reserve:5’-TGTAGACCATGTAGTTGAGGTCA-3’ |
| RIPK3 | Forward:5’-GAAGACACGGCACTCCTTGGTA-3’  Reserve:5’-CTTGAGGCAGTAGTTCTTGGTGG-3’ |
| MLKL | Forward:5’-CTGAGGGAACTGCTGGATAGAG-3’  Reserve:5’-CGAGGAAACTGGAGCTGCTGAT-3’ |
| TNF | Forward:5’-GGTGCCTATGTCTCAGCCTCTT-3’  Reserve:5’-GCCATAGAACTGATGAGAGGGAG-3’ |
| ICAM1 | Forward:5’-GTGATGCTCAGGTATCCATCCA-3’  Reserve:5’-CACAGTTCTCAAAGCACAGCG-3’ |
| CXCL2 | Forward:5’-CATCCAGAGCTTGAGTGTGACG-3’  Reserve:5’-GGCTTCAGGGTCAAGGCAAACT-3’ |
